# Epitopes in the HA and NA of H5 and H7 avian influenza viruses that are important for antigenic drift

**DOI:** 10.1093/femsre/fuae014

**Published:** 2024-05-11

**Authors:** Jasmina M Luczo, Erica Spackman

**Affiliations:** Australian Animal Health Laboratory, Australian Centre for Disease Preparedness, Commonwealth Scientific and Industrial Research Organisation, East Geelong, Victoria 3219, Australia; Exotic & Emerging Avian Viral Diseases Research, Southeast Poultry Research Laboratory, United States National Poultry Research Center, Agricultural Research Service, United States Department of Agriculture, Athens, GA 30605, United States

**Keywords:** H5, H7, avian influenza virus, haemagglutinin, neuraminidase, antigenic drift, immune escape mutants

## Abstract

Avian influenza viruses evolve antigenically to evade host immunity. Two influenza A virus surface glycoproteins, the haemagglutinin and neuraminidase, are the major targets of host immunity and undergo antigenic drift in response to host pre-existing humoral and cellular immune responses. Specific sites have been identified as important epitopes in prominent subtypes such as H5 and H7, which are of animal and public health significance due to their panzootic and pandemic potential. The haemagglutinin is the immunodominant immunogen, it has been extensively studied, and the antigenic reactivity is closely monitored to ensure candidate vaccine viruses are protective. More recently, the neuraminidase has received increasing attention for its role as a protective immunogen. The neuraminidase is expressed at a lower abundance than the haemagglutinin on the virus surface but does elicit a robust antibody response. This review aims to compile the current information on haemagglutinin and neuraminidase epitopes and immune escape mutants of H5 and H7 highly pathogenic avian influenza viruses. Understanding the evolution of immune escape mutants and the location of epitopes is critical for identification of vaccine strains and development of broadly reactive vaccines that can be utilized in humans and animals.

## Teaser

This review provides a comprehensive overview of the antigenic evolution of the haemagglutinin and neuraminidase of H5 and H7 avian influenza viruses.

## Introduction

Influenza A virus (IAV) haemagglutinin (HA) and neuraminidase (NA) continue to evolve antigenically to evade recognition by the host immune response. The two major types of antigenic evolution of IAV are antigenic shift and antigenic drift (Webster et al. 1992), which are driven by selection of escape mutants by antibody and cellular immunity. Antigenic shift occurs when the HA gene of a circulating virus is replaced by a novel HA gene segment (genetic shift), negating the ability of the host immune response to recognize the antigenically novel HA. Antigenic shift can arise from *in toto* infection of a novel influenza virus or from reassortment events in co-infected cells. Antigenic drift describes the accumulation of mutations in the HA and/or NA genes that cause amino acid changes that enable IAV to evade host immunity. As part of pandemic preparedness frameworks, the genetic and antigenic evolution of highly pathogenic avian influenza viruses (HPAIVs) is closely monitored to ensure that HPAI candidate vaccine viruses (CVVs) are protective against circulating viruses, and to inform the selection of updated CVVs if necessary (World Health Organization 2022).

**Figure 1.**
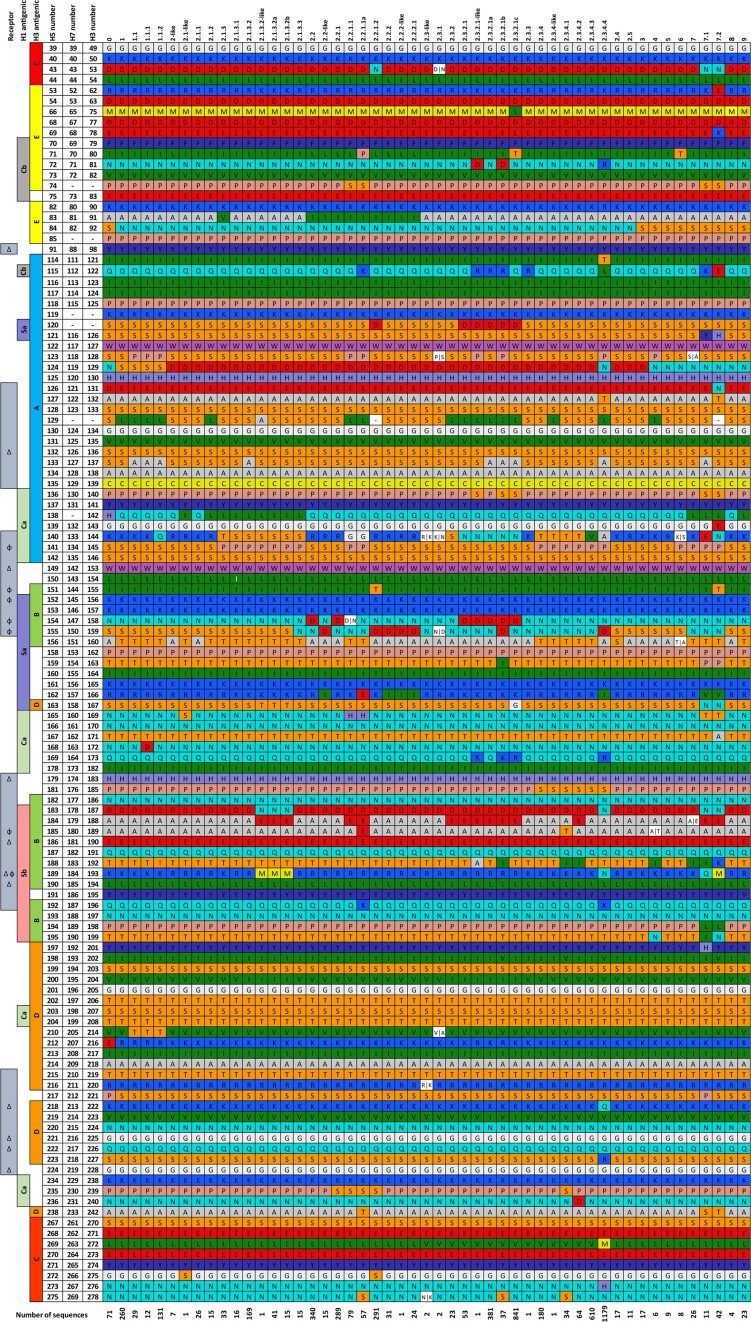
Consensus clade and subclade-specific amino acid residues are present at key antigenic sites of naturally occurring gs/Gd-lineage H5 HPAIVs. Protein sequences were downloaded from Influenza Research Database (Zhang et al. 2017), and sequences with ambiguous base calls, laboratory-generated, and duplicate sequences were omitted (*n* = 4971). Receptor column: (1) shaded rows indicate residues surrounding receptor binding site (Yang et al. 2016), (2) delta (∆) symbol indicates residues crucial to receptor specificity (Stevens et al. 2006), and (3) phi (Φ) symbol indicates residues crucial to H3 antigenic cluster transitions (Koel et al. 2013). H1 (Ca, Cb, Sa, Sb) and H3 (A–E) HA1 antigenic regions are indicated (Yang et al. 2016). Finally, H3, H5, and H7 numbering for each residue is indicated.

**Figure 2.**
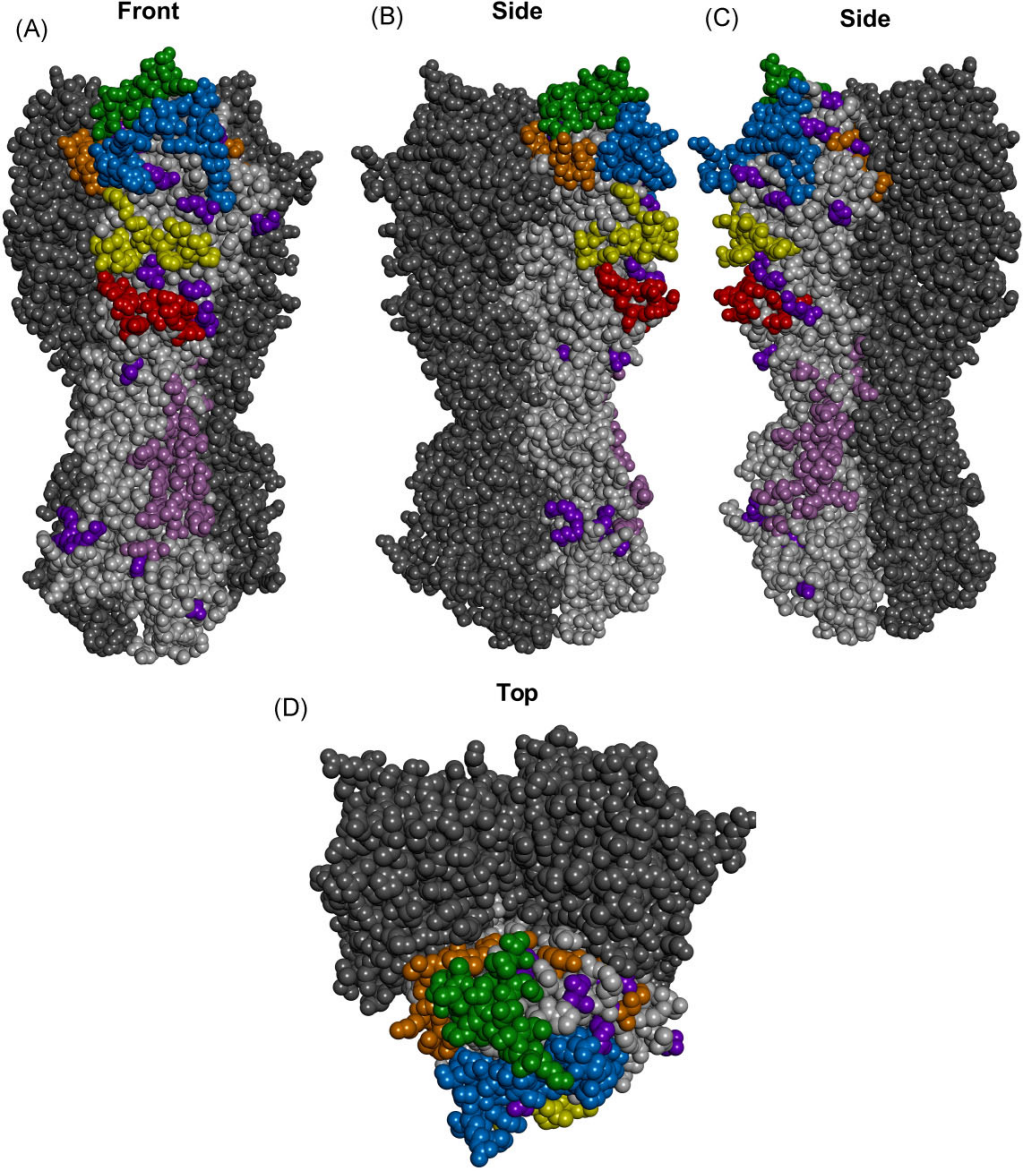
Protein homology model of H5 HPAIV haemagglutinin. Protein homology model of A/goose/Guangdong/1/1996 (H5N1, clade 0) haemagglutinin (NCBI: YP_308669.1) based on the crystal structure of 6PCX (Antanasijevic et al. 2020) was generated using SWISS-MODEL (Waterhouse et al. 2018) and modified using BIOVIA Discovery Studio (Dassault Systèmes). Trimeric gs/Gd HA with H3 antigenic sites A–E shown. Antigenic site A, blue; antigenic site B, green; antigenic site C, red; antigenic site D, orange; antigenic site E, yellow; HA stem antigenic residues targeted by two or more bnAbs, light purple; miscellaneous immune escape mutations, dark purple. (A) Front view. (B) Side view. (B) Side view rotated 180°. (D) Top view.

The HA glycoprotein structure is composed of a membrane distal globular head, the membrane proximal stem domain, flexible linker, transmembrane domain, and cytoplasmic tail (Wilson et al. 1981, Benton et al. 2018). The receptor-binding domain (RBD) is located within the HA global head. Structural features of the HA RBD include the and 130-loop, 150-loop, 190-helix, and 220-loop (Wu and Wilson 2020). Five antigenic regions on the globular head of H3 HA (antigenic sites A–E) were identified as primary targets of the host antibody response (Wiley et al. 1981). Analogous sites were subsequently identified on H5 (antigenic sites 1–5) (Philpott et al. 1989) and H1 HAs [antigenic sites Sb, Sa, Ca (which includes subsites Ca_1_ and Ca_2_), and Cb] (Gerhard et al. 1981, Caton et al. 1982) (Figs 1 and 2). Work with H3 and H1 seasonal influenza has informed our understanding of antigenic sites the H5 and H7 HAs and avian influenza NAs.

*In silico* studies have revealed numerous H5 and H7 HA residues that are under positive selection pressure in avian hosts (Kosakovsky Pond et al. 2008, Duvvuri et al. 2009, Xiong et al. 2019) (Table S1). Additional work examining positive selection pressure of human origin H5 HPAIVs identified similar amino acid subsites to those selected in avian systems (Duvvuri et al. 2009). Most of these residues cluster in antigenic sites A and B, and to a lesser extent, antigenic site D, suggesting that antigenic sites A and B are predominately targeted by the host immune response and likely to play a crucial role in the antigenic evolution of avian influenza virus (AIV). Interestingly, some of the codons encoding these residues are under positive selection pressure in human H3 IAVs (Bush et al. 1999), suggesting that these sites are broadly integral to the ability of IAVs evade host immunity. Antigenic drift and associated antigenic cluster transitions of human H3 viruses from 1968 to 2003 were associated with seven residues located adjacent to the RBD in antigenic site A, residue 145, and antigenic site B, residues 155, 156, 158, 159, 189, and 193 (H3 numbering) (Koel et al. 2013). Of these seven residues, amino acids 145, 158, 159, and 193 (H3 numbering; H5: 141, 154, 155, and 189) have been identified as being under positive selection in H5 AIVs (Duvvuri et al. 2009).

The other major surface glycoprotein, NA, is a tetrameric glycoprotein with a membrane distal head domain, membrane proximal stalk domain, transmembrane domain, and cytoplasmic domain (Varghese et al. 1983). NA features numerous structural loops, namely, the 150-loop (residues 147–152), the 270-loop (residues 267–276), 340-loop (residues 342–347), and the 430-loop (residues 429–433) (Sun et al. 2014). NA catalytic function is attributed to R118, D151, R152, R224, E276, R292, R371, and Y406 (N2 numbering), with Y406 being the key nucleophilic residue (Vavricka et al. 2013). Residues E119, R156, W178, S179, D/N198, I222, E227, E277, D293, and E425 form a scaffold that stabilize the residues crucial for catalytic function (reviewed in Shtyrya et al. 2009). The NA catalytic site and rim, and outside of the catalytic site constitute two major antigenic regions. Within these regions, seven variable segments were described, I-VII, (Colman et al. 1983). Subsequently, Webster et al. proposed four antigenic regions (1–4) of which variable segments I–IV described by Colman et al. map to antigenic region 2 (Webster et al. 1984). Mapping of the remaining variable segments V–VII to antigenic regions 1, 3, or 4 proposed by Webster et al. is not in the available literature, as such, this review will refer to NA variable segments. Antigenic epitopes on NA are less well described compared to HA epitopes. Similarly, NA residues under selection pressure have been identified *in silico* (from start methionine: 211, 338, 382, 389; N2 numbering: 210, 340, 385, 393; N1 numbering: 196, 323, 367, 374) (Kosakovsky Pond et al. 2008).

The first antigenic evolution study to experimentally characterize antigenic drift was first described in 1950 (Archetti and Horsfall 1950). To examine residues crucial to the antigenicity of H5 and H7 AIVs, experimentally derived HA and NA antigenic variants have been generated by *in vitro* or *in ovo* selection of escape mutants in the presence of monoclonal antibodies (mAbs) (Lentz et al. 1984, Air et al. 1985, 1990, Webster et al. 1987, Philpott et al. 1989, Saito et al. 1994, Kaverin et al. 2002, 2007, Chen et al. 2009, Krylov et al. 2009, Ferreira et al. 2010, Prabakaran et al. 2010, Rudneva et al. 2010, He and Kwang 2013, Zhu et al. 2013, Itoh et al. 2014, Kobayashi-Ishihara et al. 2014, Henry Dunand et al. 2015, Kaverin et al. 2015, Tan et al. 2015, Claes et al. 2016, Henry Dunand et al. 2016, Thornburg et al. 2016, Wan et al. 2016, Gronsang et al. 2017, Ohkawara et al. 2017, Ito et al. 2019, Li et al. 2019a, Okuda et al. 2019, Timofeeva et al. 2020a, Xiong et al. 2020, Strohmeier et al. 2022, Chang et al. 2023, Lyashko et al. 2024), polyclonal antiserum (Lambkin et al. 1994, Cleveland et al. 1997, Höper et al. 2012, Kalthoff et al. 2013, Sitaras et al. 2014, Chang et al. 2019, Sitaras et al. 2020), or following infection of vaccinated birds (Hinshaw et al. 1990, Beato et al. 2014, Nguyen et al. 2017). Antigenic variants that lead to evasion of the neutralizing host immune response may also be associated with changes in functional attributes, such as receptor binding dynamics (Hensley et al. 2009), pH of fusion, or thermostability. For consistency, H3 and N2 numbering is used throughout (Burke and Smith 2014, Influenza Research Database 2022), although to aid comparisons we also provide H5 and H7 (N7) numbering (Fig. 1).

## Molecular determinants of haemagglutinin antigenic drift

### Antigenic site A

Antigenic site A is also located within the solvent exposed membrane distal RBD of HA and includes amino acid residues 121–146 (H5: 114–142; H7: 111–135) (Figs 1 and 2) (Yang et al. 2016) and features the 130-loop (H3: 135–138; H5: 131–134; H7: 125–128) (Gamblin et al. 2004) of which residue 136 (H5: 132; H7: 126) directly interacts with sialic acid receptors (Ha et al. 2001) and influences receptor specificity (Martín et al. 1998).

Substitutions in antigenic site A likely play a major role in the antigenic drift of H7 AIVs. Computational analysis of Eurasian H7s antigenic epitopes revealed higher substitution rates in epitopes A and B, whereas substitutions in North American H7s were higher in epitopes B and C (Liu et al. 2015). Following natural infection of humans with H7N9 AIV, isolated mAbs predominantly bound antigenic site A or trimer interface site II, although mAbs that had haemagglutination inhibition (HI) activity predominately bound to antigenic sites A and B (Gilchuk et al. 2021). Characterization of a panel of murine mAbs raised against H7N9 AIV demonstrated that all mAbs with neutralizing activity primarily targeted antigenic site A and to a lesser extent, to antigenic sites A and D (Ito et al. 2019). Substitutions in antigenic site A also play a role in antigenic evolution of H5 AIVs, although subsites are influential, they may not be the immunodominant antigenic epitope. An early study mapping the antigenic landscape of American-lineage H5 AIV described escape mutants with substitutions in antigenic site A (R122Q, S145P), although the frequency was lower than escape mutants containing substitutions in antigenic site B (Philpott et al. 1989, 1990).

H7N9 (A/Anhui/1/2013) escape mutants capable of evading the panel of murine mAbs discussed above contained G144E (H5: G140E; H7: G133E), G144E + V505A (H5: G140E + V506A; H7: G133E + V499A), A135T + D60Y + L226Q (H5: A131T + D51Y + L222Q; H7: A125T + D50Y + L217Q), A135T + Q78R/H + L226Q (H5: A131T + Q69R/H + L222Q; H7: A125T + Q69R/H + L217Q), or A135T + S83P + L226Q (H5: A131T + S75P + L222Q; H7: A125T + S73P + L217Q) substitutions (Ito et al. 2019). The A135T + L226Q double mutation was crucial for immune escape. An H7N9 A135T (H3 numbering) escape mutant has also been described following selection using ferret polyclonal antisera (Chang et al. 2019). The A135T substitution has also been selected following H7N9 antigenic evolution studies with human mAbs (Chang et al. 2023), suggesting that this subsite is universally targeted. This substitution is present in naturally occurring isolates (Chang et al. 2019) and leads to a modest reduction in affinity to human and avian sialic acid receptor analogues, which is likely mediated by formation of an *N*-linked glycosylation (glycan shielding) (Chang et al. 2020). An American-lineage H7N2 A135S/T mutant has recently been described (Lyashko et al. 2024). Epitope masking by *N*-linked glycosylation is a well-recognized mechanism of antigenic drift (Seidel et al. 1991, Abe et al. 2004). In another study, formation of an *N*-linked glycosite in H7N9 at amino acid N133 (H5: 128; H7: 123), mediated by T135 substitution (H5: 131; H7: 125) led to epitope masking and immune escape (Alvarado-Facundo et al. 2016) and naturally occurring H7N9 viruses with 135T are able to escape vaccine induced immunity in poultry (Yin et al. 2021). Additionally, H5 and/or H7 escape mutants with substitutions at subsites 141 (Schmeisser et al. 2015, Tan et al. 2016), 143 (Kobayashi-Ishihara et al. 2014, Tan et al. 2016, Thornburg et al. 2016, Gronsang et al. 2017), 144 (discussed in detail below), 145 (discussed in detail below), or 146 (Henry Dunand et al. 2016) are frequently reported. These subsites are in the H7 antigenic motif ^140^RR-SGSS^146^ (H5 equivalent: ^140^PYQGKSS^146^), suggesting that this motif may be a hotspot for antigenic diversity. The H5 epitope ^120^KIQIIPKSSWS^128^ (H5: 113–123; H7: 110–118), which is located in the membrane distal head has also been identified as a motif for neutralizing antibody binding (Xiong et al. 2015), and H5 and H7 escape mutant harbouring substitutions at residues 120, 122, 124, 125, 125_A_, 125_B_, 126, or 128 in this epitope have been described (Philpott et al. 1989, 1990, Kaverin et al. 2007, Rudneva et al. 2010, Höper et al. 2012, He et al. 2013b, Henry Dunand et al. 2016, Nguyen et al. 2017, Ohkawara et al. 2017, Timofeeva et al. 2020a).

Although the majority of H7 antigenic evolution studies have been performed using H7N9 AIVs and human or murine mAbs, one study has examined the antigenic evolution of American-lineage H7N2 (A/turkey/New York/4550-5/1994) using chicken polyclonal antiserum. An escape mutant containing G129E substitution (plus others in antigenic sites D and E) (immature protein: G137E; H5: G124E; H7: G119E) appeared following selection with chicken polyclonal serum (Sitaras et al. 2020). Substitutions at subsite 129 have been frequently reported (Kaverin et al. 2002, He et al. 2013b, Henry Dunand et al. 2016, Timofeeva et al. 2020a) (He, immature protein: G137R; Henry Dunand, immature protein: G137E; Timofeeva, H3 numbering: D129N)—this subsite is located in a recently identified epitope that is either partly or transiently exposed on the pre-fusion conformation of HA (Turner et al. 2019). Altered receptor binding dynamics have been described for H5 escape mutants with substitutions at amino acid 129 (Ilyushina et al. 2004). Further studies selecting variants using avian polyclonal antisera would be of interest as this likely more closely reflects selection pressure in the natural host.

Amino acid 144 is influential on H5 subclade 2.3.4.4 antigenic drift (immature protein: 156; H5: 140; H7: 133) (Li et al. 2020a) and has been reported to be under positive selection pressure in H7N9 AIVs (Xiong et al. 2019). Substitutions at this site are frequently reported in H5 and H7 escape mutants. Characterization of H5 and H7 antigenic escape mutants identified amino acid 144 (H5: 140; H7: 133) as crucial for recognition by neutralizing antibodies with frequent substitutions at this subsite (Lambkin et al. 1994, Kaverin et al. 2002, Höper et al. 2012, Sitaras et al. 2014, Hervé et al. 2015, Zhang et al. 2015, Tan et al. 2016, Ito et al. 2019, Li et al. 2019a, Chang et al. 2023) and vaccine induced evolution of H7N3 virus in poultry identified two substitutions in antigenic site A, one being G144E (H5: 140; H7: 133) (Beato et al. 2014). Subsite 144 is located within the H7 antigenic motif ^140^RRSGSS^146^ (Schmeisser et al. 2015, Tan et al. 2016) (H5 equivalent: ^140^PYQGKSS^146^).

The importance of subsite 144 in the antigenic evolution of H5 AIVs and the interplay of antigenicity and receptor binding dynamics have been demonstrated by Kaverin et al. and follow-up studies characterizing phenotypic attributes of the escape mutants. An American-lineage H5N2 (A/mallard/Pennsylvania/1984 (mouse adapted)) antigenic site A escape mutants selected for using individual anti-H5 mAbs contained substitutions at N129D, D131N, R57S + D131N, D131N + K411R, and R144G (H5: N124D, D126N, R48S + D126N, D126N + K412R, and R140G; H7: N119D, D121N, R47S + D121N, D121N + K405R, and R133G) (Kaverin et al. 2002). Characterization of phenotypic effects of these H5 escape mutants determined that N129D was associated with increased affinity to 3′SLN-PAA, SiaLe^X^-PAA, and SiaLe^A^-PAA receptor analogues and R144G increased affinity to 3′SLN-PAA, SiaLe^X^-PAA, SiaLe^C^-PAA and SiaLe^A^-PAA receptor analogues. A D131N escape mutant exhibited lower affinity to 3′SLN-PAA and SiaLe^C^-PAA, likely mediated by glycan shielding (Ilyushina et al. 2004) and vaccination of mice with D131N mutant afforded lower protection against challenge with wild type virus (Smirnov et al. 2004). The formation of a glycosite by 131 N substitution in naturally occurring H5 isolates leads to immune evasion (Gu et al. 2019). Another study selecting for H5N1 A/goose/Guangdong/1/1996 (gs/Gd)-lineage (A/Vietnam/1203/2004) escape mutants using mAbs described S126Y + I155T, G143E, K144E, and S145F/P/T substitutions in antigenic site A (H5: S121Y + I151T, G139E, K140E, and S141F/P/T; H7: S116Y + I144T, G132E, K133E, and S134F/P/T (Kaverin et al. 2007). H5 escape mutants with antigenic site A substitutions at subsite 144 (H5: 140; H7: 133) (Kobayashi-Ishihara et al. 2014) and 144–147 (H5: 140–143; H7: 133–136) (Okuda et al. 2019) have subsequently been described. Importantly, H5 vaccine mismatches at amino 144 (H5: 140; H7: 133) significantly reduced protective efficacy following challenge of chickens (Criado et al. 2020) and reverse genetics studies have confirmed a crucial role of subsite 144 (H3 numbering) in mediating H5 antigenicity (Cattoli et al. 2011, Hervé et al. 2015). The frequent detection of substitutions at subsite 144 (H5: 140; H7: 133) suggests that it is likely to be a major neutralizing subsite. Substitutions at subsite 144 in antigenic site A have been selected irrespective of H5 or H7 strain or lineage, highlighting its likely importance in antigenic drift, and indeed, naturally occurring gs/Gd-lineage H5 HPAIVs exhibit substantial variation at this subsite (Fig. 1, Fig. S1).

Although amino acid 144 plays a major role in H5 and H7 antigenic drift, substitutions at amino acid 145 can also significantly affect antigenicity. A seminal antigenic epitope mapping study describing H5 escape mutants reported amino acid 145 (immature protein: 157; H5: 141; H7: 134) as a target of neutralizing antibodies (Philpott et al. 1989, 1990). Subsequently, escape mutants with substitutions at subsite 145 have been described (S145F, immature protein: S157P, immature protein: S152P, S145P/Y/del) (Krylov et al. 2009, Höper et al. 2012, Henry Dunand et al. 2016, Timofeeva et al. 2020a), the presence of Pro imparting increased thermal stability of HA (Timofeeva et al. 2020a). As amino acid 145 is implicated in major H3 antigenic cluster transitions (Koel et al. 2013) and is under positive selection in H5 AIVs (Smith et al. 2006, Kosakovsky Pond et al. 2008, Vijaykrishna et al. 2008, Duvvuri et al. 2009), it is likely that amino acid 145 plays an influential role in the antigenicity of H5 AIVs. Using reverse genetics, the role of subsite 145 in mediating H5 antigenicity has been confirmed experimentally (H5: 141; H7: 134) (Cattoli et al. 2011).

Interestingly, phenotypic attributes conferred by one amino acid substitution may not be conferred if another amino acid was selected for at the subsite. Although an American-lineage R144G (H5: R140G; H7: R133G) escape mutant described above had increased affinity to numerous α-2,3 receptor analogues, subsequent phenotypic characterization of a gs/Gd-lineage escape mutant containing the N144S substitution (H5: N140S; H7: N133S) had reduced binding to 3′SLN-PAA receptor analogue and to chicken erythrocytes, and exhibited a reduction in thermostability compared the parental strain (An et al. 2019). Another study characterizing Eurasian non-gs/Gd-lineage escape mutants containing S145P/Y substitutions (H5: 141; H7: 134) demonstrated that HA thermostability of escape mutants was dependent on this amino acid (Timofeeva et al. 2020a), and HI activity has been shown to be modulated by the amino acid present at subsite 57 (immature protein: 65) (Henry Dunand et al. 2016). These results support earlier work with H1 IAVs demonstrating that phenotypic attributes, such as antigenic escape, mediated by one amino acid may not be conferred if another amino acid is substituted at the antigenic subsite (Doud et al. 2017).

Collectively, substitutions at subsites 144 and 145 are highly influential on the antigenicity of H5 and H7 AIVs and are likely to be important subsites contributing to AIV antigenic evolution.

### Antigenic site B

Antigenic site B is located within the solvent exposed, membrane distal, RBD of HA and includes residues 155–160 (H5: 151–156; H7: 144–147 150–151), 186–194 (H5: 182–190; H7: 177–185), and 196–199 (H5: 192–195; H7: 187–190) (Figs 1 and 2). The RBD structural features, the 150-loop (H3: 155–163; H5: 151–159; H7: 144–154) (Tzarum et al. 2017) and the 190-helix (H3: 190–198; H5: 186–194; H7 181–189) (Gamblin et al. 2004) are present in antigenic site B. Three residues critical for receptor specificity are present in antigenic site B. Residue 190 (H5: 186; H7: 181) influences binding to human-type (α-2,6) sialic acid receptors (Glaser et al. 2005) and influences respiratory droplet transmission efficiency in ferrets (Tumpey et al. 2007). Residue 193 (H5: 189; H7: 184) influences affinity, but not specificity, of H5 AIV to avian-type (α-2,3) sialic acid receptors (Stevens et al. 2006, Peng et al. 2018) and residue 194 (H5: 190; H7: 185) directly interacts with sialic acid receptors (Ha et al. 2001). Although, the specificity of AIVs is more complex than ‘exclusive’ binding to one receptor type (and/or moiety) (Liu et al. 2022), as exemplified by H9N2 AIVs with α-2,6 sialic acid preference have been isolated from avian species (Matrosovich et al. 2001). Finally, 85% (6/7) of residues involved with major H3 antigenic changes are present in antigenic site B (Koel et al. 2013).

Antigenic site B seems to be a major target of the host immune response to H5 AIV. A seminal study mapping the antigenic landscape of North American-lineage H5 AIV highlighted crucial roles for antigenic sites B, and to a lesser extent A and E, in evading recognition by neutralizing antibodies (Philpott et al. 1989, 1990). Substitutions in antigenic site B included R193I (H5: 189; H7: 184), and K156E alone (immature protein: K168E; H5: K152E; H7: K145E) or in combination with substitution at amino acid 186 (K156E + A186T) (immature protein: K168E + A198T; H5: K152E + A182T; H7: K145E + A177T) (Philpott et al. 1989, 1990). Escape mutants harbouring substitutions at 186 are commonly reported (Chen et al. 2009, Henry Dunand et al. 2015, Timofeeva et al. 2020a) and have been associated with a reduction in thermal stability (Timofeeva et al. 2020a,b). H5 and H7 antigenic evolution studies consistently identify immune escape mutants with substitutions at 156 (H5: 152; H7: 145) (Kaverin et al. 2002, 2007, Chen et al. 2009, He and Kwang 2013, Timofeeva et al. 2020a) (located within the 150 loop) and 193 (H5: 189; H7: 184) (Chen et al. 2009, Ferreira et al. 2010, He et al. 2010, Ibañez et al. 2011, He and Kwang 2013, Itoh et al. 2014, Sitaras et al. 2014) (located within th e 190 helix) suggesting these subsites are crucial to immune evasion and antigenic drift. As described above, amino acids 156 and 193 (H5: 152; H7: 145 and H5: 189; H7: 184) are involved with major H3 antigenic cluster transitions (Koel et al. 2013) and likely play a pivotal role in H5 and H7 antigenicity. Additionally, subsite 156 lies within a CD8^+^ cytotoxic T lymphocyte (CTL) epitope and mutation of which abolished CTL recognition (discussed below).

H5 and H7 escape mutants frequently contain substitutions in the 150-loop (H3: 155–163; H5: 151–159; H7: 144–154). Escape mutants with substitutions at 155 (Kaverin et al. 2007, Timofeeva et al. 2020b), 156 (discussed above), 157 (Kaverin et al. 2002, Thornburg et al. 2016, Lyashko et al. 2024), 158 (Ibañez et al. 2011, Höper et al. 2012, Zhang et al. 2015, Chang et al. 2023), 158_B_ (Thornburg et al. 2016, Huang et al. 2019) (158_A_ and 158_B_ are present in H7 but not H5 AIVs), 159 (He et al. 2010, Höper et al. 2012, He and Kwang 2013), 160 (discussed below), 162 (Rudneva et al. 2010), and 163 (Paul et al. 2017) are described. Phenotypically, K156N or K157M substitutions increase affinity to α-2,3 receptor analogues (Ilyushina et al. 2004), R162K decreased the pH of fusion, R162W has no detectable effect on pH of fusion, and R162G decreased thermostability (Kaverin et al. 2015). Additionally, subsites 156, 158, and 159 are located within CTL epitopes (discussed below). Importantly, subsite 158 is associated with H3 antigenic cluster transitions (Koel et al. 2013) and 158 N also significantly contributes to H5 antigenicity by glycan shielding (Wang et al. 2010, Zhang et al. 2015).

Generation of H5 HPAIV subclade 2.3.4.4 H5N8 escape mutants using mouse anti-H5 subclade 2.3.4.4 mAbs yielded D53N and H276N, G55R, K125_A_N, and A160T substitutions (manuscript numbering: D47N and H287N, G50R, K124N, and A160T; H5: D43N and H273N, G46R, K119N, and A156T; H7: D43N and H267N, G45R, K115_A_N, and A151T). Of these, residue 160 has been identified as playing a crucial role in H5 subclade 2.3.4.4 (Ohkawara et al. 2017) and H7N9 (Yin et al. 2021) antigenicity. A naturally occurring H7N1 HPAIV (A/turkey/Italy/589/2000) with 160T was antigenically distinct from H7 AIVs with 160A and introduction of A160T into Eurasian H7N9 by site directed mutagenesis significantly reduced HI titres (Jang and Ross 2021). H7N9 and H7N2 escape mutants harbouring A160T and A160V/E substitution, respectively, have been described (Chang et al. 2019, Lyashko et al. 2024). Residue 160 is located adjacent to the RBD and is involved in major H3 antigenic cluster transitions (Koel et al. 2013) and substitutions at residue 160 can impart dual receptor binding specificity (Gu et al. 2017, Gao et al. 2018) and influence contact transmission in guinea pigs (Gu et al. 2017).

Although residue 189 is associated with H3 antigenic cluster transition (Koel et al. 2013) and is located within the putative 190 helix, escape mutants with substitutions at this position are infrequent. Nevertheless, A189E (immature protein: A201E; H5: A185E; H7: A180E) substitution has been identified in H5 and H7 escape mutants (Höper et al. 2012, Henry Dunand et al. 2015). Moreover, escape mutants with a substitutions at 185 (which flanks antigenic site B) (Rudneva et al. 2010), 187 (Kaverin et al. 2007), and 188 (Gronsang et al. 2017) are also infrequently described.

Subsites in antigenic site B have been shown to be crucial for antigenicity of 2.3.4.4 H5 HPAIVs. Specifically, the combined substitutions at antigenic subsites 193 and 196 (immature protein: 205 and 208; H5: 189 and 192; H7: 184 and 187), which are located in the 190 helix, are pivotal to antigenic drift (Li et al. 2020a), although subsites 81, 144, 227, and 276 were influential (immature protein: 88, 156, 239, and 289; H5: 72, 140, 223, 273; H7: 70, 133, 218, and 267) (Li et al. 2020a). Adjacent to subsite 196, the formation of an *N*-linked glycosite at residues 197–199 (H5: 193–195; H7: 188–190) has been shown to modulate Australian-lineage H7 HPAIV virulence, and H5 and H7 escape mutants with substitutions at subsites 198 and/or 199 have been described (Höper et al. 2012, Sitaras et al. 2014, Vasudevan et al. 2018, Lyashko et al. 2024) (Höper—immature protein: P210S and T211A; H3: P198S and T199A; H5: P194S and T195A; H7: P189S and T190A, Sitaras—H3: P198S; H5: P194S; H7: P189S, Vasudevan—H3: G198E; H5: G194E; H7: G189E, Lyashko—H3: E198G; H5: E194G; H7: E189G). However, of these mutants only P198S (A/turkey/Turkey/1/2005) yielded formation of an *N*-linked glycosite. Finally, a North American H7N2 escape mutant with a substitution at amino acid 200 (K200N—H5: 196, H7: 191), which lies between antigenic sites B and D, has been described (Lyashko et al. 2024). This amino acid lies within a recently described HA head trimer interface epitope (TI-2, described below) (Dong et al. 2020).

### Antigenic site C

Residues forming antigenic site C include 49–50, 53–54, 270–276, and 278. (H5: 39–40, 43–44, 267–273, and 275; H7: 39–40, 43–44, 261–267, and 269) (Yang et al. 2016) (Figs 1 and 2). This antigenic region does not feature RBD structural components or residues crucial for receptor specificity, though overlaps with the vestigial esterase and fusion domains of HA.

The footprint of the broadly neutralizing antibody (bnAb) MEDI8852 has been partially mapped to antigenic site C (H3 residues 54, 276, and 278; H5: 44, 273, and 275; H7: 44, 267, and 269) (Kallewaard et al. 2016), with the bnAb 39.29 light chain also interacting with residue 278 (H5: 275; H7: 269) (Nakamura et al. 2013). Antigenic escape mutants in epitope C have been described, D53N/Y, D54_A_Y, and G55E (amino acids 54_A_ and 55 flank antigenic site C) (Kobayashi-Ishihara et al. 2014), D53N + H276N (reported as D47N + H287N) (Ohkawara et al. 2017), Y274H and N278S (immature protein: Y287H and N291S) (Höper et al. 2012), and D275G (Lyashko et al. 2024), although substitutions at site C occur at a lower frequency than A and B. Additionally, substitutions at amino acid 55 and 57, which flanks antigenic site C, have been described (H7N9, G55E, R57K/W, H5N1, G55R) (Henry Dunand et al. 2015, Henry Dunand et al. 2016, Ohkawara et al. 2017). Recently, a novel conserved epitope in antigenic site C of H5 HPAIV was identified (Zhu et al. 2013). Characterization of antigenic escape mutants revealed residues 53, 83 (which is not located in antigenic site C but mapped to H1 antigenic site Cb), 274, and 276 (H5: 43, 75, 271, and 273; H7: 43, 73, 265, and 267) were critical for neutralizing antibody recognition (Zhu et al. 2013). Subsequently, another conserved epitope partially overlapping this region has been described (H3: HA^277-291^; H5: HA^274-288^; H7: HA^268-282^) (Wawegama et al. 2016). Despite a general trend for conservation in antigenic site C, some subsites are under positive selection in H5N1 viruses isolated from humans (H3: 272 and 275; H5: 269 and 272; H7: 263 and 266) (Duvvuri et al. 2009), H7 AIVs isolated up until 2012 (H3: 275; H5: 272; H7: 266) (Xiong et al. 2019) and may play a host-specific role in adaptation (H3: 276; H5: 273; H7: 267) (He et al. 2020). Moreover, compared to Eurasian H7s, North American H7s exhibit increased variation in antigenic sites C (Liu et al. 2015), possibly contributing lineage-specific antigenic differences.

### Antigenic site D

Antigenic site D is located within the RBD of HA and amino acids 167, 201–208, 214, 216–220, 222–227, and 242 (H5: 163, 197–204, 210, 212–216, 218–223, and 238; H7: 158, 192–199, 205, 207–211, 213–218, and 233) form this antigenic region (Yang et al. 2016) (Figs 1 and 2). Antigenic site D features the RDB secondary structure, the 220-loop (H3: 221–228; H5: 217–224; H7: 212–219) (Gamblin et al. 2004) and numerous residues in antigenic site D are implicated in receptor binding specificity, namely, amino acids 222, 225–226 (H5: 218, 221–222; H7: 213, 216–217) and just adjacent to antigenic site D, residue 228 (H5: 224; H7: 219). Amino acid 222 (H5: 218; H7: 213) has been shown to influence binding of IAVs to fucosylated isoforms of α-2,3 sialic acid (Xiong et al. 2013, Yang et al. 2013, Hiono et al. 2016) and substitutions at this position (K222Q) alters antigenicity and significantly reduces HI and virus neutralizing titres (Tan et al. 2015), although another study reported that K222N had no effect on antigenicity (Rudneva et al. 2010). An H7N9 escape mutant with G225D has been described (Henry Dunand et al. 2015). Amino acid 225 (H5: 221; H7: 216) is associated with dual receptor binding specificity of H1 pandemic influenza viruses (Glaser et al. 2005, Chutinimitkul et al. 2010). The presence of Q226 (H5: 222; H7: 217) in HA mediates α-2,3 sialic acid specificity of AIVs (Rogers et al. 1983), highlighting the interplay between antigenicity and receptor specificity. A Q226 L (H5: 222: H7: 217) substitution in AIVs increases binding to α-2,6 sialic acids (Matrosovich et al. 2000) and amino acid 226 in combination with other substitutions can switch receptor specificity from α-2,3 to α-2,6 sialic acids. Amino acid 226 in combination with 224 (Imai et al. 2012) or 228 (Naeve et al. 1984, Maines et al. 2011) (H5: 220 or 224; H7: 215 or 219) switch receptor binding specificity between α -2,3 and α-2,6 sialic acids. Furthermore, *in silico* analysis suggested a single substitution, G228S, was sufficient to increase H7N9 binding to α-2,3-linked and α-2,6-linked sialic acids, and lead to extensive binding of recombinant HA to human tracheal sections (Tharakaraman et al. 2013). These amino acids have also been shown to contribute to the ability of H5 to transmit by aerosol droplet in mammals (Herfst et al. 2012, Imai et al. 2012), highlighting the critical role of amino acid 226 to receptor specificity and the ability of zoonotic spillover of AIVs from avian to human hosts.

Antigenically, generation of H7N9 escape mutants revealed that amino acid 226 (H5: 222; H7: 217) is critical to antigenicity (Chang et al. 2019, 2023, Ito et al. 2019) and receptor specificity. Recent work has suggested that the L226Q mutation leads to increased avidity to sialic acids present on erythrocytes, potentially biasing HI-mediated antigenic characterization (Wang et al. 2020). Multiple studies have also revealed a role for amino acid 205 (immature protein: 214; H5: 201; H7: 196) in the antigenic evolution of H7N2 (Sitaras et al. 2020), H7N9 (Henry Dunand et al. 2015, Ito et al. 2019), and H5N1 AIVs (Höper et al. 2012). Despite not being located within the RBD, amino acid 205 has been shown to alter receptor binding preference of H3 influenza viruses from α-2,3-linked to α-2,6-linked receptor analogues (Suzuki et al. 1989).

An H5 immune escape mutant generated following passage in homologous vaccinated chickens contained G167D substitution (immature protein: 179; H5: 163; H7: 158) was associated with a reduction in HI and virus neutralizing titres (Nguyen et al. 2017). Amino acid 166 (H5: 162; H7: 157) that flanks antigenic site D (and distantly flanks antigenic site B), is under positive selection (Duvvuri et al. 2009, He et al. 2020) and numerous studies have described escape mutants with substitutions at this position (Rudneva et al. 2010, He et al. 2013a,b, Gronsang et al. 2017) or influencing cross-protective efficacy of H5 vaccines (Criado et al. 2020), suggesting a significant role for this residue in the antigenic drift of H5 and H7 AIVs. Amino acid 227 (H5: 223; H7: 218) located in the 220-loop is also under positive selection. This subsite has been shown to contribute to subclade 2.3.4.4 antigenic drift (Li et al. 2020b), and a gs/Gd-lineage H5 escape mutant with S227R substitution has been described (Tan et al. 2015). Additionally, substitutions at amino acid 230, which also flanks antigenic site D although more distantly than 166, have also been described for gs/Gd-lineage H5 escape mutants [M230T and M230I (H5: 226; H7: 221)] (Rudneva et al. 2010, Sitaras et al. 2014). Finally, gs/Gd-lineage H5 escape mutants with A242T (H5: A238T; H7: A233T) have been described (Sitaras et al. 2014).

### Antigenic site E

Antigenic site E is located within the vestigial esterase subdomain of HA and includes residues 62–63, 75, 77–82, and 90–92 (H5: 53–54, 66, 68–74, and 82–85; H7: 52–53, 65, 67–72, and 80–82) (Figs 1 and 2).

A seminal study mapping the antigenic landscape of the head domain of a North American-lineage H5N9 HA identified amino acid 62 (immature protein: 69; H5: 53; H7: 52) as a target of neutralizing antibodies (Philpott et al. 1989, 1990). There was no discernible effect on viral pathogenicity following challenge of chickens with this antigenic site E escape mutant, which is in contrast to escape mutants with substitutions in antigenic site B (Philpott et al. 1989). Immunoselection studies with H7N9 have identified Q78R/H substitutions (Ito et al. 2019). An H7N2 antigenic escape mutant selected for using polyclonal chicken antisera with a mutation at amino acid 79 (immature protein: 87; H5: 70; H7: 69) has been described (Sitaras et al. 2020). Epitope mapping of an anti-H5 mAb targeting the vestigial esterase domain revealed a role for amino acid 79 (in addition to 62 and 69) for recognition by the mAb, and mutation of this amino acid reduced mAb binding (Paul et al. 2017). Finally, gs/Gd H5N1 antigenic escape mutants with substitutions at N81D and P82_A_Q (immature protein: N88D and P90Q) have been described (Höper et al. 2012). R81 is the most frequently detected amino acid in currently circulating subclade 2.3.4.4 H5 HPAIVs (Fig. 1), although lysine, serine, and asparagine are also detected (Fig. S1), and amino acid 81 has shown to be influential in the antigenic drift of 2.3.4.4 HPAIVs (Li et al. 2020a).

### Haemagglutinin stem epitopes

More recently, antigenic regions on the HA stem recognized by bnAbs have been described (Corti et al. 2011, Dreyfus et al. 2012, Nakamura et al. 2013, Fu et al. 2016, Kallewaard et al. 2016). In the stem region of HA, epitope mapping of bnAb footprints reveals numerous residues targeted by human and murine bnAbs, including amino acids 38, 40–42, 54 (antigenic site C), 83, 276 (antigenic site C), 278 (antigenic site C), 289, 291–293, 318, 328_B_-340, 347–350, and 363–372, 374–379, 381–383, 385, and 387–389 (H5: 28, 30–32, 44, 75, 273, 275, 286, 288–290, 315, 327–341, 348–351, and 364–373, 375–380, 382–384, 386, and 388–390; H7(N7): 28, 30–32, 44, 73, 267, 269, 280, 282–284, 309, 321–334, 341–344, and 357–366, 368–373, 375–377, 379, 381–383) (Corti et al. 2011, Dreyfus et al. 2012, Nakamura et al. 2013, Fu et al. 2016, Kallewaard et al. 2016). Of these, antigenic hotspots for bnAb binding are evident at amino acids 347–350 and 363–389. A H7N9 linear epitope,^363^TAADYKSTQSAIDQITGKLN^382^, has been identified in this stem antigenic hotspot (Li et al. 2019b). Another class of bnAbs target a membrane-proximal anchor epitope. The HA anchor epitope is adjacent to the hotspot described above and includes amino acids W343, H354, Q356, S361, and Y363 (Guthmiller et al. 2022). Further research examining bnAbs elicited in avian species would be of interest, though H5 and H7 escape mutants with substitutions at amino acids E368K (Kalthoff et al. 2013) and I374T (Henry Dunand et al. 2015) in this bnAb hotspot are described.

### Haemagglutinin T cell epitopes

Although antibody mediated antigenic evolution of AIV is extensively characterized, little is known about avian T cell-mediated immunity, and most of the available data are for chickens. The CTL response is crucial for limiting IAV replication and in viral clearance (Lukacher et al. 1984). In chickens, CTL responses are believed to be important for the robustness of vectored vaccines against antigenic changes (Mo et al. 2023). Numerous H5 and H7 HA T cell epitopes have been described, some of which overlap with B cell epitopes, although, these epitopes were characterized using human or murine CTLs. For example, a CD8^+^ T cell epitope [major histocompatibility complex (MHC) class II restricted clone (Hioe and Hinshaw 1989)] located in the globular head of H5 HA^146-157^ (SFYRNVVWLIKK) (immature protein: 158–169; H5: 142–153; H7: 135–146) (Hioe et al. 1990) overlaps with B cell antigenic sites A and B. A single substitution at residue 156 (immature protein: 168; H5: 152; H7: 145) was sufficient to abolish murine CTL recognition (Hioe et al. 1990). Another MHC Class II restricted CD4 conserved epitope present on H5 AIVs is ^469^FYHKCDNECME^479^ (H5: 470–480; H7: 463–473) (Wang et al. 2021) and antigenic escape mutant at amino acid D474N (H5: 475; H7: 468) has been described (Ibañez et al. 2011). This conserved epitope is also recognized by H1, H3, H4, H6, H7, H8, H9, and H10 antisera (Wang et al. 2021).

Several studies have characterized the chicken CTL response following H5 or H7 AIV exposure (Singh et al. 2010, Schmiedeke et al. 2019, Hao et al. 2020). The first H5 epitope confirmed to be recognized by and functionally activate chicken CD4^+^ and CD8^+^ T cells is HA^234-248^ (WTILKPSDTINFESN) (immature protein: 246–260; H5 numbering: 230–244; H7: 225–239) (Haghighi et al. 2009). Subsequent work has identified H5 MHC Class I restricted epitopes polymerase acidic 123–130 (Li et al. 2020b), nucleoprotein (NP) 67–74 (Zhang et al. 2016), NP89-97, and NP198-206 (Hou et al. 2012), and numerous H7 NP- and M1-specific MHC Class I restricted epitopes (Reemers et al. 2012). The NP-specific CTL response is greater than the HA-specific CTL response (Singh et al. 2010). It is currently unclear whether these CTL epitopes are under selection pressure, particularly in wild avian species with multiple exposures to AIVs.

An epitope unique to H7 AIVs lies adjacent to antigenic site A, ^113^RESGG^117^ (H7: 103–107). This epitope is highly conserved in Eurasian-lineage H7s but not North American-lineage, highlighting diagnostic potential to differentiate between these lineages (Yao et al. 2020). Another pan-H7 epitope elicited from natural human infection with A/British Columbia/1/2015 (H7N9) has been identified in the HA head trimer interface (TI-2), with antigenic amino acids being 101, 103–104, 163, 182, 184, 199–201, 208–216, 231, and 233 and a subset of these being important for (non-neutralizing) antibody interaction (Dong et al. 2020). Another trimer interface epitope (TI-1) previously described (Turner et al. 2019) was composed of amino acids ^128^SG^129^, N157, 158_B_N, ^160^AFPQM^164, 197^SGN^199^, L201, and T246. Finally, a pH sensitive epitope located in the HA head of H7N9 AIV includes amino acids ^101^KFVNEE^106^, Q110, E114, ^209^YQQS^212^, and ^233^HWLMLN^238^ (Yu et al. 2017).

### Non-canonical haemagglutinin epitopes

Whilst most described escape mutants harbour substitutions at key antigenic regions, immune escape mutants with substitutions at non-canonical antigenic sites are described. These include an American-lineage H5 escape mutant with E46K (associated with A186T Philpott et al. 1989, 1990). H7N9 immune escape mutants with K173N and D348N (Henry Dunand et al. 2016), A149D (which flanks antigenic site A and it situated in a CD8 + T cell epitope) (Thornburg et al. 2016), V309I, R354K, I374N/T (Henry Dunand et al. 2015), and R256H (Vasudevan et al. 2018) substitutions have been described. Notably, amino acid 256 has been shown to be under positive selection in H5 viruses (Duvvuri et al. 2009). gs/Gd-lineage H5 escape mutants with (S133P)+H244R + R326G (Nguyen et al. 2017) and E368K have been reported (Kalthoff et al. 2013). Several escape mutants with substitutions in the HA cleavage site, the predominant marker of AIV virulence (Luczo et al. 2018), have been described including R326G (Nguyen et al. 2017), and K327Q and T328K (Lyashko et al. 2024)—through ampliative reasoning, T328K may be reversion of a mouse-adapted AIV back to the wild type sequence upon propagation in embryonated chicken eggs.

### Molecular determinants of neuraminidase antigenic drift

Whilst the HA glycoprotein is the predominant protective antigen, NA is receiving increasing attention as a protective immunogen. Vaccination of chickens with NA can elicit complete protection against H5 HPAIV challenge (Webster et al. 1988), and mucosal NA immunity has been shown to prevent IAV transmission (McMahon et al. 2019). A deletion in the NA stalk, a known marker of poultry adaptation (Matrosovich et al. 1999) does not affect NA antigenicity (Els et al. 1985). NA antigenic epitopes and immune escape mutants are not as well described compared to HA epitopes. Here, we expand our focus to escape mutants generated using avian isolates or isolates that harbour avian-origin NA due to the ability of H5 and H7 AIV to reassort with numerous NA subtypes. N2 numbering is used throughout.

### Variable segment I

**Figure 3.**
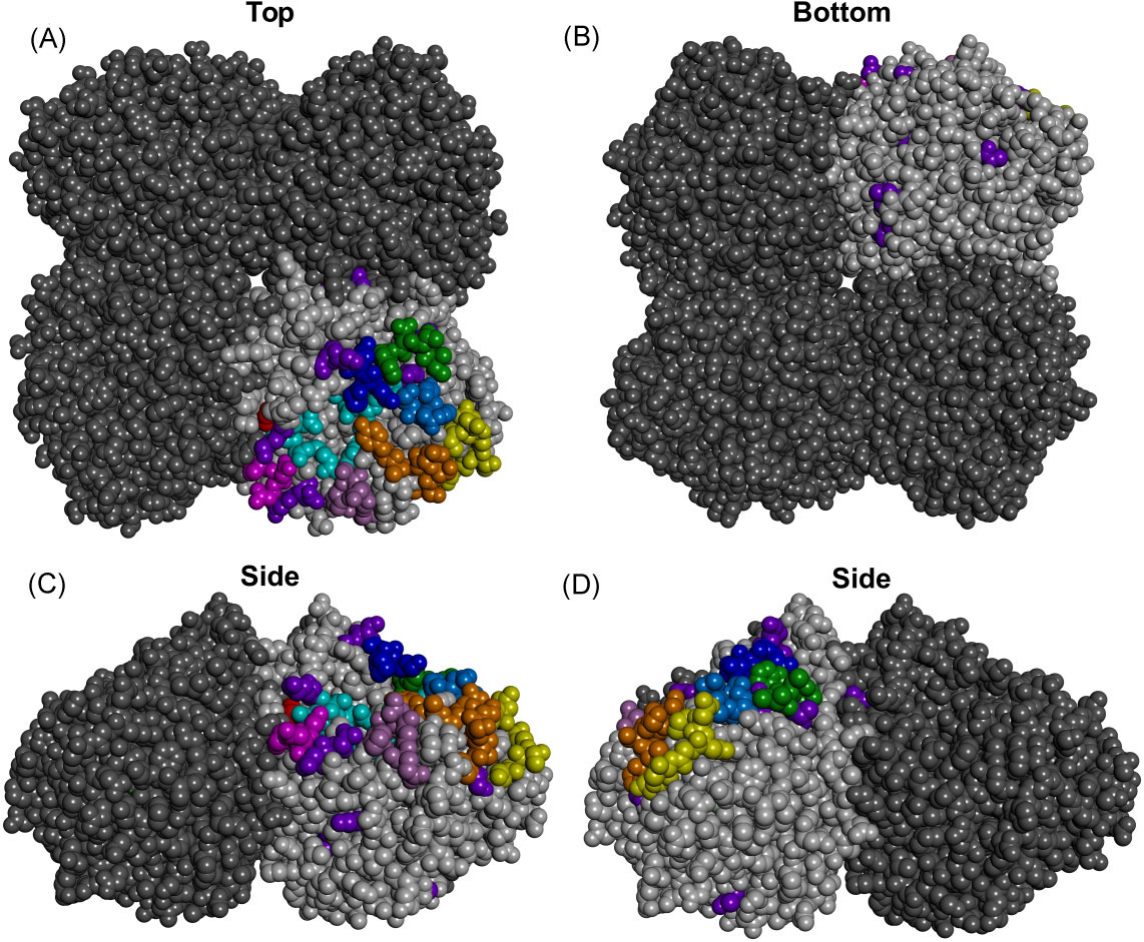
Protein homology model of N1 HPAIV neuraminidase. Protein homology model of A/Viet Nam/1203/2004 (H5N1, clade 1) neuraminidase (NCBI: ADD97097.1) based on the crystal structure of 5HUG (H5N1, subclade 2.3.4.4) (Yang et al. 2016) was generated using SWISS-MODEL (Waterhouse et al. 2018) and modified using BIOVIA Discovery Studio (Dassault Systèmes). Tetrameric gs/Gd-lineage NA with variable segments I-VII shown. Variable segment I, yellow; variable segment II, orange; variable segment III, light blue; variable segment IV, green; variable segment V, dark blue; variable segment VI, magenta; variable segment VII, red; additional variable segment, light purple; miscellaneous immune escape mutations, dark purple. (A) Top view. (B) Bottom view. (C) Side view. (D) Side view rotated 90°.

Variable segment I is located on the lateral solvent exposed surface of NA (Fig. 3, yellow residues) and includes residues 328–336. An early study examining pandemic N2 escape mutants (of which the NA is of avian origin) reported D329N, and N334S + K368E in variable region I following selection using mAbs (Air et al. 1985). Additionally, selection of escape mutant with N329D has been described for Australian-lineage N9 isolate (Webster et al. 1987).

### Variable segment II

Variable segment II is located adjacent to variable region I on the lateral plane of NA (Fig. 3, orange residues). Variable segment II is composed of residues 339–347 and incorporates the 340-loop (residues 342–347). Several N2 escape mutants with substitutions at residue 344 are described. These include pandemic N2 with R344I (Laver et al. 1982), R344K/G/I/T/S (Lentz et al. 1984), and R344G/K (Air et al. 1985). N9 immune escape mutants from the Yangtze River Delta-lineage ((H7)N9) with N345S and N347S (Xiong et al. 2020) and N8 escape mutants with N344K and G346R/E (Saito et al. 1994) substitutions have been mapped to this variable segment. An N2 escape mutant with R338S substitution is described (Wan et al. 2016), suggesting that amino acid 338, which flanks variable segment II, is also a part of variable segment II epitope. Functional consequence of substitutions in variable segment II include alterations to NA thermostability (Laver et al. 1982).

### Variable segment III

Variable segment III is located on the top solvent exposed surface of NA and incorporates amino acids 367–370 (Fig. 3, light blue residues) and features loop 1 of the second sialic binding site, also called the hemabsorbing (HB) site (amino acids 367, 370, and 372) (Varghese et al. 1997). The NC-41 epitope partially maps to variable segment III (amino acids 368–372) (Colman et al. 1987).

A multitude of immune escape mutants map to variable segment III. Immune escape mutants with substitutions at amino acid 367 include N8 escape mutants with S367N (Saito et al. 1994), N9 escape mutants with S367N (Webster et al. 1987), or S367N/G/R (Air et al. 1990), and an (H7)N9 immune escape mutant with S367P (Xiong et al. 2020). Notably, glycan shielding seems to be a mechanism of immune evasion at this subsite.

Escape mutants with substitutions at amino acid 368 are frequently described. They include a N2 escape mutants with K368E alone (Laver et al. 1982) or in in combination with N334S (K368E + N334S) (Air et al. 1985). N9 escape mutants with substitutions at this position include I368R (Webster et al. 1987) and (H7)N9 immune escape mutant with T368 L (Xiong et al. 2020).

N9 escape mutants commonly report substitutions at amino acid 369. These include an N9 escape mutant with A369D (Webster et al. 1987) and an (H7)N9 immune escape mutant with A369T (Xiong et al. 2020). N2 and N9/(H7)N9 escape mutants with S370 L are commonly reported (Air et al. 1985, Webster et al. 1987, Xiong et al. 2020). Amino acid 372 flanks variable segment III and numerous AIV NA escape mutants with substitutions at this position are reported (Webster et al. 1987, Air et al. 1990), suggesting that this amino acid is part of the variable segment III epitope. Escape mutants with substitutions at this position include N9 escape mutants with S372Y (Webster et al. 1987) or S372F (Air et al. 1990).

### Variable segment IV

Variable segment IV is located on the top surface exposed surface of NA (Fig. 3, green residues) and includes residues that form loop 2 of the HB site (Varghese et al. 1997). The NC-41 epitope partially maps to variable region IV (amino acids 400–403) (Colman et al. 1987). Mapping of immune escape mutants suggests that residue 399 also forms the variable segment IV epitope.

Immune escape mutants with substitution in this variable segment include N8 escape mutants with D399N and N400K (Saito et al. 1994), N9 escape mutants also with N400K (Webster et al. 1987, Air et al. 1990), (H7)N9 escape mutant with A401G and W403 L (Xiong et al. 2020), and N2 escape mutant with W403R (Air et al. 1985).

### Variable segment V

Variable segment V is located on the top solvent exposed surface of NA (Fig. 3, dark blue residues) and is comprised of amino acids 431–434. Adjacent to variable segment V are variable segments III and IV. The 430-loop is located within variable segment V, and it also features loop 3 of the second sialic acid binding site (HB site) (Varghese et al. 1997). The NC-41 epitope partially maps to variable region IV (amino acids 430–434) (Colman et al. 1987).

N9 escape mutants with K432N (Webster et al. 1987) or K432E + K435G (Air et al. 1990) have been described. N9/(H7)N9 escape mutants with substitutions at amino acids 435 and 436, both of which flank variable segment V, suggest that these residues also form part of variable segment V epitope. In addition to the N9 escape mutant with a substitution at amino acid 435 described above, (H7)N9 escape mutants with D435E and K436R have been reported (Xiong et al. 2020). Finally, a human N1 escape mutant with R430Q suggests that amino acid 430, which flanks variable segment V, also likely forms part of variable segment V epitope.

### Variable segment VI

Variable segment VI is located on the lateral surface of NA, along the rim of the active site, and close to a protomer:protomer interface (Fig. 3B, pink residues). Variable segment VI includes amino acids 197–199, and the Mem5 Fab footprint maps these amino acids (Venkatramani et al. 2006). Residue 198 forms part of the dimensional structure of the active site (reviewed in (Shtyrya et al. 2009), and numerous escape mutants with substitutions at these sites have been reported. These include (H5)N1 gs/Gd-lineage escape mutant with D198E (Nguyen et al. 2017), N2 immune escape mutants with D198N and K199N (Wan et al. 2016), N8 immune escape mutant with S199P (Saito et al. 1994), and an (H7)N9 escape mutant with N198S (Xiong et al. 2020).

Amino acids 220 and 221 flank variable segment VI and are likely part of the same epitope, and the Mem5 epitope includes these amino acids (Venkatramani et al. 2006). Several immune escape mutants at these positions have been reported, including N6 escape mutant with G220E that was associated with a strong reduction in NI activity (Strohmeier et al. 2022) and an N9 escape mutant with R220Q (Webster et al. 1987). A N2 immune escape mutant with D221H substitution has been described (Laver et al. 1982).

### Variable segment VII

Variable segment VII is located on top of NA, along the rim of the active site, and close to a protomer:protomer interface (Fig. 3, red residues). Colman et al. described only amino acid 153 as situated in this variable segment. To date, human N1 immune escape mutant with a substitution at this position (S153I) (Yasuhara et al. 2018), but not avian, have been described. However, numerous avian immune escape mutants with substitution at amino acid 150, which flanks 153, are described. These include an N2 immune escape mutant with H150Q/N (Air et al. 1985), an N8 escape mutant with K150E (Saito et al. 1994), and an (H7)N9 immune escape mutant with H150P (Xiong et al. 2020). It is likely that amino acids 150 and 153 belong to the same epitope. Notably, the Mem5 Fab footprint maps to amino acid 150 within variable segment VII, and amino acids 147 and 154 that flank variable segment VII (Venkatramani et al. 2006), suggesting that the epitope may be larger than initially described. The universally conserved Asn146 glycosite is located near variable segment VII, the absence of which is associated with H1N1 neurovirulence (Li et al. 1993) and altered N8 substrate preference (Saito and Kawano 1997).

### Other neuraminidase epitopes

In addition to the seven variable segments described by Colman et al. (1983), analysis of antigenic escape mutants suggests the presence of another epitope (Fig. 3, light purple residues) situated on the NA lateral surface and surrounding the active site. This additional epitope includes amino acids 245–249 (N1: 231–235). Recent work with N6 and (H7)N9 AIVs has mapped numerous substitutions in antigenic escape mutants to this region. Specifically, N6 escape mutants contained P245Q, N248S, or R249G/K substitutions in this region. N248S resulted in a significant reduction in neuraminidase inhibition (NI) activity and P246Q and R250G/K resulted in a complete loss of NI activity (Strohmeier et al. 2022). The Mem5 Fab footprint maps to amino acids 249 and 251 in this region (Venkatramani et al. 2006). Additionally, a study examining (H7)N9 escape mutants identified A246G and T247N substitutions in this region (Xiong et al. 2020).

Several immune escape mutants with substitutions located on the bottom surface of NA have been described (Fig. 3B). An early study examining N2 antigenic drift described an escape mutant with R253S substitution (Lentz et al. 1984). Escape mutants with substitutions located on the underside of NA include an Eurasian-lineage (H5)N3 escape mutant with I257M (Timofeeva et al. 2020a), a gs/Gd-lineage (H5)N1 immune escape mutant with R130K and T187A (Höper et al. 2012), and an N8 escape mutant with N284G (Saito et al. 1994). Substitutions located underneath NA have also been described for human N2 (Kirkpatrick Roubidoux et al. 2021), suggesting that this region is targeted by both avian and mammalian hosts.

Substitutions located between the promoter interface have been described, including a gs/Gd-lineage (H5)N1 immune escape mutant with R107K substitution (Höper et al. 2012). Targeting of the NA active site of numerous AIVs in, including (H5)N1, N2, N4, N5, N6, N7, (H5)N8, and (H7)N9 by the bnAbs 1G01, 1E01, and 1G04, has recently been described (Stadlbauer et al. 2019). Finally, amino acids 222–230 constitute the only conserved epitope across all NA subtypes. Modification to this epitope impacts viral fitness as evidenced by lower viral growth, altered substrate binding, reduced enzymatic activity, and lower NA thermostability (Doyle et al. 2013).

## Conclusions

H5 and H7 AIVs continue to evolve antigenically to evade the host immune response. Experimental studies that have generated antigenically advanced immune escape mutants have provided crucial insights to key antigenic epitopes on haemagglutinin and neuraminidase proteins. Within the HA glycoprotein, H7 escape mutants are frequently detected with substitutions in antigenic site A, and in H5 escape mutants antigenic site B seems to play a major role in the antigenic drift of contemporary isolates. Moreover, the contribution of NA immunity is gaining increased recognition of its importance in anti-IAV immunity, although to date, remains understudied. Studies examining the functional fitness of antigenically advanced escape mutants have provided further insights as to why certain escape mutations may be selected and the broad landscape of potential mutations among different lineages. Understanding the molecular determinants of antigenic drift of both HA and NA is crucial to the development of broadly protective vaccines to combat the threat of H5 and H7 AIVs. Finally, in addition to preserving food security and maintaining domestic and wild animal health, increasing research efforts to understand the emergence, evolution, and fitness of antigenically novel strains is crucial to understanding risk and pandemic potential.

## Supplementary Material

fuae014_Supplemental_File

## References

[bib1] Abe Y, Takashita E, Sugawara K et al. Effect of the addition of oligosaccharides on the biological activities and antigenicity of influenza A/H3N2 virus hemagglutinin. J Virol. 2004;78:9605–11. 15331693 10.1128/JVI.78.18.9605-9611.2004 PMC514993

[bib2] Air GM, Els MC, Brown LE et al. Location of antigenic sites on the three-dimensional structure of the influenza N2 virus neuraminidase. Virology. 1985;145:237–48. 2411049 10.1016/0042-6822(85)90157-6

[bib3] Air GM, Laver WG, Webster RG. Mechanism of antigenic variation in an individual epitope on influenza virus N9 neuraminidase. J Virol. 1990;64:5797–803. 1700825 10.1128/jvi.64.12.5797-5803.1990 PMC248733

[bib4] Alvarado-Facundo E, Vassell R, Schmeisser F et al. Glycosylation of residue 141 of subtype H7 influenza A hemagglutinin (HA) affects HA-pseudovirus infectivity and sensitivity to site A neutralizing antibodies. PLoS One. 2016;11:e0149149. 26862918 10.1371/journal.pone.0149149 PMC4749315

[bib5] An S-H, Lee C-Y, Hong S-M et al. Novel mutations evading avian immunity around the receptor binding site of the clade 2.3.2.1c hemagglutinin gene reduce viral thermostability and mammalian pathogenicity. Viruses. 2019;11:923. 31600990 10.3390/v11100923 PMC6832455

[bib6] Antanasijevic A, Durst MA, Lavie A et al. Identification of a pH sensor in Influenza hemagglutinin using X-ray crystallography. J Struct Biol. 2020;209:107412. 31689502 10.1016/j.jsb.2019.107412 PMC7111647

[bib7] Archetti I, Horsfall FL Jr. Persistent antigenic variation of influenza A viruses after incomplete neutralization in ovo with heterologous immune serum. J Exp Med. 1950;92:441–62. 14778924 10.1084/jem.92.5.441 PMC2135986

[bib8] Beato MS, Xu Y, Long L-P et al. Antigenic and genetic evolution of low-pathogenicity avian influenza viruses of subtype H7N3 following heterologous vaccination. Clin Vaccine Immunol. 2014;21:603–12. 24554694 10.1128/CVI.00647-13 PMC4018894

[bib9] Benton DJ, Nans A, Calder LJ et al. Influenza hemagglutinin membrane anchor. Proc Natl Acad Sci USA. 2018;115:10112–7. 30224494 10.1073/pnas.1810927115 PMC6176637

[bib10] Burke DF, Smith DJ. A recommended numbering scheme for influenza A HA subtypes. PLoS One. 2014;9:e112302. 25391151 10.1371/journal.pone.0112302 PMC4229193

[bib11] Bush RM, Fitch WM, Bender CA et al. Positive selection on the H3 hemagglutinin gene of human influenza virus A. Mol Biol Evol. 1999;16:1457–65. 10555276 10.1093/oxfordjournals.molbev.a026057

[bib12] Caton AJ, Brownlee GG, Yewdell JW et al. The antigenic structure of the influenza virus A/PR/8/34 hemagglutinin (H1 subtype). Cell. 1982;31:417–27. 6186384 10.1016/0092-8674(82)90135-0

[bib13] Cattoli G, Milani A, Temperton N et al. Antigenic drift in H5N1 avian influenza virus in poultry is driven by mutations in major antigenic sites of the hemagglutinin molecule analogous to those for human influenza virus. J Virol. 2011;85:8718–24. 21734057 10.1128/JVI.02403-10 PMC3165837

[bib14] Chang P, Lukosaityte D, Sealy JE et al. Antigenic characterization of human monoclonal antibodies for therapeutic use against H7N9 avian influenza virus. J Virol. 2023;97:e01431–01422. 36541801 10.1128/jvi.01431-22 PMC9888198

[bib15] Chang P, Sealy JE, Sadeyen J-R et al. Amino acid residue 217 in the hemagglutinin glycoprotein is a key mediator of avian influenza H7N9 virus antigenicity. J Virol. 2019;93:e01627–01618. 30282714 10.1128/JVI.01627-18 PMC6288333

[bib16] Chang P, Sealy JE, Sadeyen J-R et al. Immune escape adaptive mutations in the H7N9 avian influenza hemagglutinin protein increase virus replication fitness and decrease pandemic potential. J Virol. 2020;94:e00216–00220. 32699084 10.1128/JVI.00216-20 PMC7495387

[bib17] Chen Y, Qin K, Wu WL et al. Broad cross-protection against H5N1 avian influenza virus infection by means of monoclonal antibodies that map to conserved viral epitopes. J Infect Dis. 2009;199:49–58. 19032063 10.1086/594374

[bib18] Chutinimitkul S, Herfst S, Steel J et al. Virulence-associated substitution D222G in the hemagglutinin of 2009 pandemic influenza A(H1N1) virus affects receptor binding. J Virol. 2010;84:11802–13. 20844044 10.1128/JVI.01136-10 PMC2977876

[bib19] Claes F, Morzaria SP, Donis RO. Emergence and dissemination of clade 2.3.4.4 H5Nx influenza viruses—how is the Asian HPAI H5 lineage maintained. Curr Opin Virol. 2016;16:158–63. 26991931 10.1016/j.coviro.2016.02.005

[bib20] Cleveland SM, Taylor HP, Dimmock NJ. Selection of neutralizing antibody escape mutants with type A influenza virus HA-specific polyclonal antisera: possible significance for antigenic drift. Epidemiol Infect. 1997;118:149–54. 9129591 10.1017/s0950268896007303 PMC2808776

[bib21] Colman PM, Laver WG, Varghese JN et al. Three-dimensional structure of a complex of antibody with influenza virus neuraminidase. Nature. 1987;326:358–63. 2436051 10.1038/326358a0

[bib22] Colman PM, Varghese JN, Laver WG. Structure of the catalytic and antigenic sites in influenza virus neuraminidase. Nature. 1983;303:41–4. 6188957 10.1038/303041a0

[bib23] Corti D, Voss J, Gamblin SJ et al. A neutralizing antibody selected from plasma cells that binds to group 1 and group 2 influenza A hemagglutinins. Science. 2011;333:850–6. 21798894 10.1126/science.1205669

[bib24] Criado MF, Sá e Silva M, Lee D-H et al. Cross-protection by inactivated H5 prepandemic vaccine seed strains against diverse goose/Guangdong lineage H5N1 highly pathogenic avian influenza viruses. J Virol. 2020;94:e00720–00720. 32999029 10.1128/JVI.00720-20 PMC7925181

[bib25] Dong J, Gilchuk I, Li S et al. Anti–influenza H7 human antibody targets antigenic site in hemagglutinin head domain interface. J Clin Invest. 2020;130:4734–9. 32749241 10.1172/JCI136032 PMC7456233

[bib26] Doud MB, Hensley SE, Bloom JD. Complete mapping of viral escape from neutralizing antibodies. PLOS Path. 2017;13:e1006271. 10.1371/journal.ppat.1006271 PMC5363992 28288189

[bib27] Doyle TM, Jaentschke B, Van Domselaar G et al. The universal epitope of influenza A viral neuraminidase fundamentally contributes to enzyme activity and viral replication. J Biol Chem. 2013;288:18283–9. 23645684 10.1074/jbc.M113.468884 PMC3689970

[bib28] Dreyfus C, Laursen NS, Kwaks T et al. Highly conserved protective epitopes on influenza B viruses. Science. 2012;337:1343–8. 22878502 10.1126/science.1222908 PMC3538841

[bib29] Duvvuri VRSK, Duvvuri B, Cuff WR et al. Role of positive selection pressure on the evolution of H5N1 hemagglutinin. Genom Proteom Bioinf. 2009;7:47–56. 10.1016/S1672-0229(08)60032-7 PMC5054228 19591791

[bib30] Els MC, Air GM, Murti KG et al. An 18-amino acid deletion in an influenza neuraminidase. Virology. 1985;142:241–7. 4060573 10.1016/0042-6822(85)90332-0

[bib31] Ferreira HL, Lambrecht B, van Borm S et al. Identification of a dominant epitope in the hemagglutinin of an Asian highly pathogenic avian influenza H5N1 clade 1 virus by selection of escape mutants. Avian Dis. 2010;54:565–71. 20521695 10.1637/8750-033009-ResNote.1

[bib32] Fu Y, Zhang Z, Sheehan J et al. A broadly neutralizing anti-influenza antibody reveals ongoing capacity of haemagglutinin-specific memory B cells to evolve. Nat Commun. 2016;7:12780. 27619409 10.1038/ncomms12780 PMC5027281

[bib33] Gamblin SJ, Haire LF, Russell RJ et al. The structure and receptor binding properties of the 1918 influenza hemagglutinin. Science. 2004;303:1838–42. 14764886 10.1126/science.1093155

[bib34] Gao R, Gu M, Liu K et al. T160A mutation-induced deglycosylation at site 158 in hemagglutinin is a critical determinant of the dual receptor binding properties of clade 2.3.4.4 H5NX subtype avian influenza viruses. Vet Microbiol. 2018;217:158–66. 29615249 10.1016/j.vetmic.2018.03.018

[bib35] Gerhard W, Yewdell J, Frankel ME et al. Antigenic structure of influenza virus haemagglutinin defined by hybridoma antibodies. Nature. 1981;290:713–7. 6163993 10.1038/290713a0

[bib36] Gilchuk IM, Bangaru S, Kose N et al. Human antibody recognition of H7N9 influenza virus HA following natural infection. JCI Insight. 2021;6:e152403. 34437301 10.1172/jci.insight.152403 PMC8525637

[bib37] Glaser L, Stevens J, Zamarin D et al. A single amino acid substitution in 1918 influenza virus hemagglutinin changes receptor binding specificity. J Virol. 2005;79:11533–6. 16103207 10.1128/JVI.79.17.11533-11536.2005 PMC1193621

[bib38] Gronsang D, Bui AN, Trinh DQ et al. Characterization of cross-clade monoclonal antibodies against H5N1 highly pathogenic avian influenza virus and their application to the antigenic analysis of diverse H5 subtype viruses. Arch Virol. 2017;162:2257–69. 28405766 10.1007/s00705-017-3350-0

[bib39] Gu C, Zeng X, Song Y et al. Glycosylation and an amino acid insertion in the head of hemagglutinin independently affect the antigenic properties of H5N1 avian influenza viruses. Sci China Life Sci. 2019;62:76–83. 30515728 10.1007/s11427-018-9439-0

[bib40] Gu M, Li Q, Gao R et al. The T160A hemagglutinin substitution affects not only receptor binding property but also transmissibility of H5N1 clade 2.3.4 avian influenza virus in guinea pigs. Vet Res. 2017;48:7. 28166830 10.1186/s13567-017-0410-0 PMC5294818

[bib41] Guthmiller JJ, Han J, Utset HA et al. Broadly neutralizing antibodies target a hemagglutinin anchor epitope. Nature. 2022;602:314–20. 34942633 10.1038/s41586-021-04356-8 PMC8828479

[bib42] Ha Y, Stevens DJ, Skehel JJ et al. X-ray structures of H5 avian and H9 swine influenza virus hemagglutinins bound to avian and human receptor analogs. Proc Natl Acad Sci USA. 2001;98:11181–6. 11562490 10.1073/pnas.201401198 PMC58807

[bib43] Haghighi HR, Read LR, Haeryfar SMM et al. Identification of a dual-specific T cell epitope of the hemagglutinin antigen of an H5 avian influenza virus in chickens. PLoS One. 2009;4:e7772. 19901990 10.1371/journal.pone.0007772 PMC2770124

[bib44] Hao X, Li S, Chen L et al. Establishing a multicolor flow cytometry to characterize cellular immune response in chickens following H7N9 avian influenza virus infection. Viruses. 2020;12:1396. 33291218 10.3390/v12121396 PMC7762099

[bib45] He F, Kumar SR, Syed Khader SM et al. Effective intranasal therapeutics and prophylactics with monoclonal antibody against lethal infection of H7N7 influenza virus. Antiviral Res. 2013a;100:207–14. 23954322 10.1016/j.antiviral.2013.08.003

[bib47] He F, Prabakaran M, Tan Y et al. Development of dual-function ELISA for effective antigen and antibody detection against H7 avian influenza virus. BMC Microbiol. 2013b;13:219. 24083616 10.1186/1471-2180-13-219 PMC4015598

[bib46] He F, Kwang J. Monoclonal antibody targeting neutralizing epitope on H5N1 influenza virus of clade 1 and 0 for specific H5 quantification. Influenza Res Treat. 2013;2013:360675. 23533740 10.1155/2013/360675 PMC3603295

[bib48] He F, Soejoedono RD, Murtini S et al. Complementary monoclonal antibody-based dot ELISA for universal detection of H5 avian influenza virus. BMC Microbiol. 2010;10:330. 21192824 10.1186/1471-2180-10-330 PMC3023680

[bib49] He W-T, Wang L, Zhao Y et al. Adaption and parallel evolution of human-isolated H5 avian influenza viruses. J Infect. 2020;80:630–8. 32007525 10.1016/j.jinf.2020.01.012

[bib51] Henry Dunand CJ, Leon Paul E, Huang M et al. Both neutralizing and non-neutralizing human H7N9 influenza vaccine-induced monoclonal antibodies confer protection. Cell Host Microbe. 2016;19:800–13. 27281570 10.1016/j.chom.2016.05.014 PMC4901526

[bib50] Henry Dunand CJ, Leon PE, Kaur K et al. Preexisting human antibodies neutralize recently emerged H7N9 influenza strains. J Clin Invest. 2015;125:1255–68. 25689254 10.1172/JCI74374 PMC4362269

[bib52] Hensley SE, Das SR, Bailey AL et al. Hemagglutinin receptor binding avidity drives influenza A virus antigenic drift. Science. 2009;326:734–6. 19900932 10.1126/science.1178258 PMC2784927

[bib53] Herfst S, Schrauwen EJA, Linster M et al. Airborne transmission of influenza A/H5N1 virus between ferrets. Science. 2012;336:1534–41. 22723413 10.1126/science.1213362 PMC4810786

[bib54] Hervé P-L, Lorin V, Jouvion G et al. Addition of N-glycosylation sites on the globular head of the H5 hemagglutinin induces the escape of highly pathogenic avian influenza A H5N1 viruses from vaccine-induced immunity. Virology. 2015;486:134–45. 26433051 10.1016/j.virol.2015.08.033

[bib55] Hinshaw VS, Sheerar MG, Larsen D. Specific antibody responses and generation of antigenic variants in chickens immunized against a virulent avian influenza virus. Avian Dis. 1990;34:80–6. 1690984

[bib56] Hioe CE, Dybdahl-Sissoko N, Philpott M et al. Overlapping cytotoxic T-lymphocyte and B-cell antigenic sites on the influenza virus H5 hemagglutinin. J Virol. 1990;64:6246–51. 1700833 10.1128/jvi.64.12.6246-6251.1990 PMC248799

[bib57] Hioe CE, Hinshaw VS. Induction and activity of class II-restricted, Lyt-2+ cytolytic T lymphocytes specific for the influenza H5 hemagglutinin. J Immunol. 1989;142:2482–8. 2466897

[bib58] Hiono T, Okamatsu M, Igarashi M et al. Amino acid residues at positions 222 and 227 of the hemagglutinin together with the neuraminidase determine binding of H5 avian influenza viruses to sialyl Lewis X. Arch Virol. 2016;161:307–16. 26542967 10.1007/s00705-015-2660-3 PMC5030063

[bib59] Höper D, Kalthoff D, Hoffmann B et al. Highly pathogenic avian influenza virus subtype H5N1 escaping neutralization: more than HA variation. J Virol. 2012;86:1394–404. 22090121 10.1128/JVI.00797-11 PMC3264362

[bib60] Hou Y, Guo Y, Wu C et al. Prediction and identification of T cell epitopes in the H5N1 influenza virus nucleoprotein in chicken. PLoS One. 2012;7:e39344. 22745738 10.1371/journal.pone.0039344 PMC3379973

[bib61] Huang K-YA, Rijal P, Jiang H et al. Structure–function analysis of neutralizing antibodies to H7N9 influenza from naturally infected humans. Nat Microbiol. 2019;4:306–15. 30478290 10.1038/s41564-018-0303-7

[bib62] Ibañez LI, De Filette M, Hultberg A et al. Nanobodies with in vitro neutralizing activity protect mice against H5N1 influenza virus infection. J Infect Dis. 2011;203:1063–72. 21450996 10.1093/infdis/jiq168

[bib63] Ilyushina NA, Rudneva IA, Gambaryan AS et al. Receptor specificity of H5 influenza virus escape mutants. Virus Res. 2004;100:237–41. 15019242 10.1016/j.virusres.2003.12.032

[bib64] Imai M, Watanabe T, Hatta M et al. Experimental adaptation of an influenza H5 HA confers respiratory droplet transmission to a reassortant H5 HA/H1N1 virus in ferrets. Nature. 2012;486:420–8. 22722205 10.1038/nature10831 PMC3388103

[bib65] Influenza Research Database . HA subtype numbering conversion. 2022; https://www.fludb.org/brc/haNumbering.spg?method=ShowCleanInputPage&decorator=influenza (23 September 2022, date last accessed).

[bib66] Ito M, Yamayoshi S, Murakami K et al. Characterization of mouse monoclonal antibodies against the HA of A(H7N9) influenza virus. Viruses. 2019;11:149. 30754701 10.3390/v11020149 PMC6410113

[bib67] Itoh Y, Yoshida R, Shichinohe S et al. Protective efficacy of passive immunization with monoclonal antibodies in animal models of H5N1 highly pathogenic avian influenza virus infection. PLOS Path. 2014;10:e1004192. 10.1371/journal.ppat.1004192 PMC4055766 24945244

[bib68] Jang H, Ross TM. Hemagglutination inhibition (HAI) antibody landscapes after vaccination with H7Nx virus like particles. PLoS One. 2021;16:e0246613. 33735274 10.1371/journal.pone.0246613 PMC7971484

[bib69] Kallewaard Nicole L, Corti D, Collins Patrick J et al. Structure and function analysis of an antibody recognizing all influenza A subtypes. Cell. 2016;166:596–608. 27453466 10.1016/j.cell.2016.05.073 PMC4967455

[bib70] Kalthoff D, Röhrs S, Höper D et al. Truncation and sequence shuffling of segment 6 generate replication-competent neuraminidase-negative influenza H5N1 viruses. J Virol. 2013;87:13556–68. 24109212 10.1128/JVI.02244-13 PMC3838276

[bib71] Kaverin NV, Rudneva IA, Govorkova EA et al. Epitope mapping of the hemagglutinin molecule of a highly pathogenic H5N1 influenza virus by using monoclonal antibodies. J Virol. 2007;81:12911–7. 17881439 10.1128/JVI.01522-07 PMC2169086

[bib72] Kaverin NV, Rudneva IA, Ilyushina NA et al. Structure of antigenic sites on the haemagglutinin molecule of H5 avian influenza virus and phenotypic variation of escape mutants. J Gen Virol. 2002;83:2497–505. 12237433 10.1099/0022-1317-83-10-2497

[bib73] Kaverin NV, Rudneva IA, Timofeeva TA et al. Pleiotropic effects of amino acid substitutions in H5 hemagglutinin of influenza A escape mutants. Virus Res. 2015;210:81–89. 26220479 10.1016/j.virusres.2015.07.016

[bib74] Kirkpatrick Roubidoux E, McMahon M, Manuel Carreño J et al. Identification and characterization of novel Antibody epitopes on the N2 neuraminidase. mSphere. 2021;6:e00958–20. 33568453 10.1128/mSphere.00958-20 PMC8544889

[bib75] Kobayashi-Ishihara M, Takahashi H, Ohnishi K et al. Broad cross-reactive epitopes of the H5N1 influenza virus identified by murine antibodies against the A/Vietnam/1194/2004 hemagglutinin. PLoS One. 2014;9:e99201. 24945805 10.1371/journal.pone.0099201 PMC4063728

[bib76] Koel BF, Burke DF, Bestebroer TM et al. Substitutions near the receptor binding site determine major antigenic change during influenza virus evolution. Science. 2013;342:976–9. 24264991 10.1126/science.1244730

[bib77] Kosakovsky Pond SL, Poon AFY, Leigh Brown AJ et al. A maximum likelihood method for detecting directional evolution in protein sequences and its application to influenza A virus. Mol Biol Evol. 2008;25:1809–24. 18511426 10.1093/molbev/msn123 PMC2515872

[bib78] Krylov PS, Rudneva IA, Timofeeva TA et al. Amino acid substitutions in the hemagglutinin of H5 influenza virus changing the antigenic specificty and virus virulence. Vopr Virusol. 2009;54:14–9. 19882897

[bib79] Lambkin R, McLain L, Jones SE et al. Neutralization escape mutants of type A influenza virus are readily selected by antisera from mice immunized with whole virus: a possible mechanism for antigenic drift. J Gen Virol. 1994;75:3493–502. 7527838 10.1099/0022-1317-75-12-3493

[bib80] Laver WG, Air GM, Webster RG et al. Amino acid sequence changes in antigenic variants of type A influenza virus N2 neuraminidase. Virology. 1982;122:450–60. 6183823 10.1016/0042-6822(82)90244-6

[bib81] Lentz MR, Air GM, Laver WG et al. Sequence of the neuraminidase gene of influenza virus A/Tokyo/3/67 and previously uncharacterized monoclonal variants. Virology. 1984;135:257–65. 6203216 10.1016/0042-6822(84)90135-1

[bib82] Li J, Gu M, Liu K et al. Amino acid substitutions in antigenic region B of hemagglutinin play a critical role in the antigenic drift of subclade 2.3.4.4 highly pathogenic H5NX influenza viruses. Transbound Emerg Dis. 2020a;67:263–75. 31484213 10.1111/tbed.13347

[bib83] Li M, Chen L, Wang Q et al. A cross-reactive human monoclonal antibody targets the conserved H7 antigenic site A from fifth wave H7N9-infected humans. Antiviral Res. 2019a;170:104556. 31299269 10.1016/j.antiviral.2019.104556

[bib84] Li S, Schulman J, Itamura S et al. Glycosylation of neuraminidase determines the neurovirulence of influenza A/WSN/33 virus. J Virol. 1993;67:6667–73. 8411368 10.1128/jvi.67.11.6667-6673.1993 PMC238105

[bib85] Li X, Zhang L, Liu Y et al. Structures of the MHC-I molecule BF2*1501 disclose the preferred presentation of an H5N1 virus-derived epitope. J Biol Chem. 2020b;295:5292–306. 32152225 10.1074/jbc.RA120.012713 PMC7170506

[bib86] Li Z, Wan Z, Li T et al. A novel linear epitope crossing group 1 and group 2 influenza A viruses located in the helix A of HA2 derived from H7N9. Vet Microbiol. 2019b;228:39–44. 30593378 10.1016/j.vetmic.2018.11.002

[bib87] Liu M, Huang LZX, Smits AA et al. Human-type sialic acid receptors contribute to avian influenza A virus binding and entry by hetero-multivalent interactions. Nat Commun. 2022;13:4054. 35831293 10.1038/s41467-022-31840-0 PMC9279479

[bib88] Liu M, Song T, Hua S et al. Computational analysis of antigenic epitopes of avian influenza A (H7N9) viruses. Sci China Life Sci. 2015;58:687–93. 26100010 10.1007/s11427-015-4886-4

[bib89] Luczo JM, Tachedjian M, Harper JA et al. Evolution of high pathogenicity of H5 avian influenza virus: haemagglutinin cleavage site selection of reverse-genetics mutants during passage in chickens. Sci Rep. 2018;8:11518. 30068964 10.1038/s41598-018-29944-z PMC6070550

[bib90] Lukacher AE, Braciale VL, Braciale TJ. In vivo effector function of influenza virus-specific cytotoxic T lymphocyte clones is highly specific. J Exp Med. 1984;160:814–26. 6206190 10.1084/jem.160.3.814 PMC2187390

[bib91] Lyashko AV, Timofeeva TA, Rudneva IA et al. Antigenic architecture of the H7N2 influenza virus hemagglutinin belonging to the North American lineage. Int J Mol Sci. 2024;25:212. 10.3390/ijms25010212 PMC10779424 38203384

[bib92] Maines TR, Chen L-M, Van Hoeven N et al. Effect of receptor binding domain mutations on receptor binding and transmissibility of avian influenza H5N1 viruses. Virology. 2011;413:139–47. 21397290 10.1016/j.virol.2011.02.015 PMC5470842

[bib93] Martín J, Wharton SA, Lin YP et al. Studies of the binding properties of influenza hemagglutinin receptor-site mutants. Virology. 1998;241:101–11. 9454721 10.1006/viro.1997.8958

[bib94] Matrosovich M, Tuzikov A, Bovin N et al. Early alterations of the receptor-binding properties of H1, H2, and H3 avian influenza virus hemagglutinins after their introduction into mammals. J Virol. 2000;74:8502–12. 10954551 10.1128/jvi.74.18.8502-8512.2000 PMC116362

[bib95] Matrosovich M, Zhou N, Kawaoka Y et al. The surface glycoproteins of H5 influenza viruses isolated from humans, chickens, and wild aquatic birds have distinguishable properties. J Virol. 1999;73:1146–55. 9882316 10.1128/jvi.73.2.1146-1155.1999 PMC103935

[bib96] Matrosovich MN, Krauss S, Webster RG. H9N2 influenza A viruses from poultry in Asia have human virus-like receptor specificity. Virology. 2001;281:156–62. 11277689 10.1006/viro.2000.0799

[bib97] McMahon M, Kirkpatrick E, Stadlbauer D et al. Mucosal immunity against neuraminidase prevents influenza B virus transmission in guinea pigs. mBio. 2019;10:e00560–00519. 31113896 10.1128/mBio.00560-19 PMC6529633

[bib98] Mo J, Spackman E, Swayne DE. Prediction of highly pathogenic avian influenza vaccine efficacy in chickens by comparison of in vitro and in vivo data: a meta-analysis and systematic review. Vaccine. 2023;41:5507–17. 37537093 10.1016/j.vaccine.2023.07.076

[bib99] Naeve CW, Hinshaw VS, Webster RG. Mutations in the hemagglutinin receptor-binding site can change the biological properties of an influenza virus. J Virol. 1984;51:567–9. 6748165 10.1128/jvi.51.2.567-569.1984 PMC254476

[bib100] Nakamura G, Chai N, Park S et al. An *in vivo* human-plasmablast enrichment technique allows rapid identification of therapeutic influenza A antibodies. Cell Host Microbe. 2013;14:93–103. 23870317 10.1016/j.chom.2013.06.004

[bib101] Nguyen LT, Nishi T, Shichinohe S et al. Selection of antigenic variants of an H5N1 highly pathogenic avian influenza virus in vaccinated chickens. Virology. 2017;510:252–61. 28756116 10.1016/j.virol.2017.07.030

[bib102] Ohkawara A, Okamatsu M, Ozawa M et al. Antigenic diversity of H5 highly pathogenic avian influenza viruses of clade 2.3.4.4 isolated in Asia. Microbiol Immunol. 2017;61:149–58. 28370432 10.1111/1348-0421.12478

[bib103] Okuda M, Yamayoshi S, Uraki R et al. Subclade 2.2.1-specific human monoclonal antibodies that recognize an epitope in antigenic site A of influenza A(H5) virus HA detected between 2015 and 2018. Viruses. 2019;11:321. 30987023 10.3390/v11040321 PMC6521261

[bib104] Paul SS, Mok C-K, Mak T-M et al. A cross-clade H5N1 influenza A virus neutralizing monoclonal antibody binds to a novel epitope within the vestigial esterase domain of hemagglutinin. Antiviral Res. 2017;144:299–310. 28633988 10.1016/j.antiviral.2017.06.012

[bib105] Peng W, Bouwman KM, McBride R et al. Enhanced human-type receptor binding by ferret-transmissible H5N1 with a K193T mutation. J Virol. 2018;92:e02016–02017. 29491160 10.1128/JVI.02016-17 PMC5923085

[bib106] Philpott M, Easterday BC, Hinshaw VS. Neutralizing epitopes of the H5 hemagglutinin from a virulent avian influenza virus and their relationship to pathogenicity. J Virol. 1989;63:3453–8. 2473218 10.1128/jvi.63.8.3453-3458.1989 PMC250921

[bib107] Philpott M, Hioe C, Sheerar M et al. Hemagglutinin mutations related to attenuation and altered cell tropism of a virulent avian influenza A virus. J Virol. 1990;64:2941–7. 2335822 10.1128/jvi.64.6.2941-2947.1990 PMC249478

[bib108] Prabakaran M, He F, Meng T et al. Neutralizing epitopes of influenza virus hemagglutinin: target for the development of a universal vaccine against H5N1 lineages. J Virol. 2010;84:11822–30. 20844051 10.1128/JVI.00891-10 PMC2977865

[bib109] Reemers SSN, van Haarlem DA, Sijts AJAM et al. Identification of novel avian influenza virus derived CD8+ T-cell epitopes. PLoS One. 2012;7:e31953. 22384112 10.1371/journal.pone.0031953 PMC3285639

[bib110] Rogers GN, Paulson JC, Daniels RS et al. Single amino acid substitutions in influenza haemagglutinin change receptor binding specificity. Nature. 1983;304:76–8. 6191220 10.1038/304076a0

[bib111] Rudneva IA, Kushch AA, Masalova OV et al. Antigenic epitopes in the hemagglutinin of Qinghai-type influenza H5N1 virus. Viral Immunol. 2010;23:181–7. 20373998 10.1089/vim.2009.0086

[bib112] Saito T, Kawano K. Loss of glycosylation at Asn144 alters the substrate preference of the N8 influenza A virus neuraminidase. J Vet Med Sci. 1997;59:923–6. 9362042 10.1292/jvms.59.923

[bib113] Saito T, Taylor G, Laver WG et al. Antigenicity of the N8 influenza A virus neuraminidase: existence of an epitope at the subunit interface of the neuraminidase. J Virol. 1994;68:1790–6. 7509002 10.1128/jvi.68.3.1790-1796.1994 PMC236640

[bib114] Schmeisser F, Vasudevan A, Verma S et al. Antibodies to antigenic site A of influenza H7 hemagglutinin provide protection against H7N9 challenge. PLoS One. 2015;10:e0117108. 25629172 10.1371/journal.pone.0117108 PMC4309539

[bib115] Schmiedeke JK, Hoffmann D, Hoffmann B et al. Establishment of adequate functional cellular immune response in chicks is age dependent. Avian Dis. 2019;64:69–79. 10.1637/0005-2086-64.1.69 32267127

[bib116] Seidel W, Künkel F, Geisler B et al. Intraepidemic variants of influenza virus H3 hemagglutinin differing in the number of carbohydrate side chains. Arch Virol. 1991;120:289–96. 1958130 10.1007/BF01310484

[bib117] Shtyrya YA, Mochalova LV, Bovin NV. Influenza virus neuraminidase: structure and function. Acta Naturae. 2009;1:26–32. 22649600 PMC3347517

[bib118] Singh S, Briles WE, Lupiani B et al. Avian influenza viral nucleocapsid and hemagglutinin proteins induce chicken CD8+ memory T lymphocytes. Virology. 2010;399:231–8. 20116819 10.1016/j.virol.2009.12.029 PMC7111969

[bib119] Sitaras I, Kalthoff D, Beer M et al. Immune escape mutants of highly pathogenic avian influenza H5N1 selected using polyclonal sera: identification of key amino acids in the HA protein. PLoS One. 2014;9:e84628. 24586231 10.1371/journal.pone.0084628 PMC3934824

[bib120] Sitaras I, Spackman E, de Jong MCM et al. Selection and antigenic characterization of immune-escape mutants of H7N2 low pathogenic avian influenza virus using homologous polyclonal sera. Virus Res. 2020;290:198188. 33045306 10.1016/j.virusres.2020.198188

[bib121] Smirnov YA, Gitelman AK, Govorkova EA et al. Influenza H5 virus escape mutants: immune protection and antibody production in mice. Virus Res. 2004;99:205–8. 14749187 10.1016/j.virusres.2003.11.012

[bib122] Smith GJD, Naipospos TSP, Nguyen TD et al. Evolution and adaptation of H5N1 influenza virus in avian and human hosts in Indonesia and Vietnam. Virology. 2006;350:258–68. 16713612 10.1016/j.virol.2006.03.048

[bib123] Stadlbauer D, Zhu X, McMahon M et al. Broadly protective human antibodies that target the active site of influenza virus neuraminidase. Science. 2019;366:499–504. 31649200 10.1126/science.aay0678 PMC7105897

[bib124] Stevens J, Blixt O, Tumpey TM et al. Structure and receptor specificity of the hemagglutinin from an H5N1 influenza virus. Science. 2006;312:404–10. 16543414 10.1126/science.1124513

[bib125] Strohmeier S, Amanat F, Carreño JM et al. Monoclonal antibodies targeting the influenza virus N6 neuraminidase. Front Immunol. 2022;13:944907. 35967389 10.3389/fimmu.2022.944907 PMC9363587

[bib126] Sun X, Li Q, Wu Y et al. Structure of influenza virus N7: the last piece of the neuraminidase “jigsaw” puzzle. J Virol. 2014;88:9197–207. 24899180 10.1128/JVI.00805-14 PMC4136277

[bib127] Suzuki Y, Kato H, Naeve CW et al. Single-amino-acid substitution in an antigenic site of influenza virus hemagglutinin can alter the specificity of binding to cell membrane-associated gangliosides. J Virol. 1989;63:4298–302. 2476569 10.1128/jvi.63.10.4298-4302.1989 PMC251045

[bib128] Tan GS, Leon PE, Albrecht RA et al. Broadly-reactive neutralizing and non-neutralizing antibodies directed against the H7 influenza virus hemagglutinin reveal divergent mechanisms of protection. PLOS Path. 2016;12:e1005578. 10.1371/journal.ppat.1005578 PMC4833315 27081859

[bib129] Tan Y, Ng Q, Jia Q et al. A novel humanized antibody neutralizes H5N1 influenza virus via two different mechanisms. J Virol. 2015;89:3712–22. 25609802 10.1128/JVI.03014-14 PMC4403434

[bib130] Tharakaraman K, Jayaraman A, Raman R et al. Glycan receptor binding of the influenza A virus H7N9 hemagglutinin. Cell. 2013;153:1486–93. 23746830 10.1016/j.cell.2013.05.034 PMC3746546

[bib131] Thornburg NJ, Zhang H, Bangaru S et al. H7N9 influenza virus neutralizing antibodies that possess few somatic mutations. J Clin Invest. 2016;126:1482–94. 26950424 10.1172/JCI85317 PMC4811156

[bib132] Timofeeva TA, Rudneva IA, Sadykova GK et al. Variability of nonpathogenic influenza virus H5N3 under immune pressure. Acta Virol. 2020a;64:480–9. 33151742 10.4149/av_2020_415

[bib133] Timofeeva TA, Sadykova GK, Lomakina NF et al. The effect of I155T, K156Q, K156E and N186K mutations in hemagglutinin on the virulence and reproduction of influenza A/H5N1 viruses. Mol Biol. 2020b;54:861–9. 33424035 10.1134/S0026893320060126 PMC7783499

[bib134] Tumpey TM, Maines TR, Van Hoeven N et al. A two-amino acid change in the hemagglutinin of the 1918 influenza virus abolishes transmission. Science. 2007;315:655–9. 17272724 10.1126/science.1136212

[bib135] Turner HL, Pallesen J, Lang S et al. Potent anti-influenza H7 human monoclonal antibody induces separation of hemagglutinin receptor-binding head domains. PLOS Biol. 2019;17:e3000139. 30716060 10.1371/journal.pbio.3000139 PMC6375650

[bib136] Tzarum N, de Vries RP, Peng W et al. The 150-loop restricts the host specificity of human H10N8 influenza virus. Cell Rep. 2017;19:235–45. 28402848 10.1016/j.celrep.2017.03.054 PMC5452617

[bib137] Varghese JN, Colman PM, van Donkelaar A et al. Structural evidence for a second sialic acid binding site in avian influenza virus neuraminidases. Proc Natl Acad Sci USA. 1997;94:11808–12. 9342319 10.1073/pnas.94.22.11808 PMC23599

[bib138] Varghese JN, Laver WG, Colman PM. Structure of the influenza virus glycoprotein antigen neuraminidase at 2.9 Å resolution. Nature. 1983;303:35–40. 6843658 10.1038/303035a0

[bib139] Vasudevan A, Woerner A, Schmeisser F et al. Potency determination of inactivated H7 influenza vaccines using monoclonal antibody-based ELISA and biolayer interferometry assays. Influenza Other Resp Viruses. 2018;12:250–8. 10.1111/irv.12528 PMC5820428 29152878

[bib140] Vavricka CJ, Liu Y, Kiyota H et al. Influenza neuraminidase operates via a nucleophilic mechanism and can be targeted by covalent inhibitors. Nat Commun. 2013;4:1491. 23422659 10.1038/ncomms2487

[bib141] Venkatramani L, Bochkareva E, Lee JT et al. An epidemiologically significant epitope of a 1998 human influenza virus neuraminidase forms a highly hydrated interface in the NA–antibody complex. J Mol Biol. 2006;356:651–63. 16384583 10.1016/j.jmb.2005.11.061

[bib142] Vijaykrishna D, Bahl J, Riley S et al. Evolutionary dynamics and emergence of panzootic H5N1 influenza viruses. PLOS Path. 2008;4:e1000161. 10.1371/journal.ppat.1000161 PMC2533123 18818732

[bib143] Wan Z, Ye J, Sang J et al. Identification of amino acids in H9N2 influenza virus neuraminidase that are critical for the binding of two mouse monoclonal antibodies. Vet Microbiol. 2016;187:58–63. 27066709 10.1016/j.vetmic.2016.03.011

[bib144] Wang Q, Sun Z, Li J et al. Identification of a universal antigen epitope of influenza A virus using peptide microarray. BMC Vet Res. 2021;17:22. 33413356 10.1186/s12917-020-02725-5 PMC7792037

[bib145] Wang W, Lu B, Zhou H et al. Glycosylation at 158 N of the hemagglutinin protein and receptor binding specificity synergistically affect the antigenicity and immunogenicity of a live attenuated H5N1 A/Vietnam/1203/2004 vaccine virus in ferrets. J Virol. 2010;84:6570–7. 20427525 10.1128/JVI.00221-10 PMC2903256

[bib146] Wang Y, Lv Y, Niu X et al. L226Q mutation on influenza H7N9 virus hemagglutinin increases receptor-binding avidity and leads to biased antigenicity evaluation. J Virol. 2020;94:e00667–00620. 32796071 10.1128/JVI.00667-20 PMC7527056

[bib147] Waterhouse A, Bertoni M, Bienert S et al. SWISS-MODEL: homology modelling of protein structures and complexes. Nucleic Acids Res. 2018;46:W296–303. 29788355 10.1093/nar/gky427 PMC6030848

[bib148] Wawegama NK, Tarigan S, Indriani R et al. Evaluation of a conserved HA274–288 epitope to detect antibodies to highly pathogenic avian influenza virus H5N1 in Indonesian commercial poultry. Avian Pathol. 2016;45:478–92. 27009612 10.1080/03079457.2016.1167276

[bib149] Webster RG, Air GM, Metzger DW et al. Antigenic structure and variation in an influenza virus N9 neuraminidase. J Virol. 1987;61:2910–6. 3612957 10.1128/jvi.61.9.2910-2916.1987 PMC255818

[bib150] Webster RG, Bean WJ, Gorman OT et al. Evolution and ecology of influenza A viruses. Microbiol Rev. 1992;56:152–79. 1579108 10.1128/mr.56.1.152-179.1992 PMC372859

[bib151] Webster RG, Brown LE, Laver WG. Antigenic and biological characterization of influenza virus neuraminidase (N2) with monoclonal antibodies. Virology. 1984;135:30–42. 6203218 10.1016/0042-6822(84)90114-4

[bib152] Webster RG, Reay PA, Laver WG. Protection against lethal influenza with neuraminidase. Virology. 1988;164:230–7. 2452514 10.1016/0042-6822(88)90640-x

[bib153] Wiley DC, Wilson IA, Skehel JJ. Structural identification of the antibody-binding sites of Hong Kong influenza haemagglutinin and their involvement in antigenic variation. Nature. 1981;289:373–8. 6162101 10.1038/289373a0

[bib154] Wilson IA, Skehel JJ, Wiley DC. Structure of the haemagglutinin membrane glycoprotein of influenza at 3 Å resolution. Nature. 1981;289:366–73. 7464906 10.1038/289366a0

[bib155] World Health Organization . Antigenic and genetic characteristics of zoonotic influenza A viruses and development of candidate vaccine viruses for pandemic preparedness. 2022. https://cdn.who.int/media/docs/default-source/influenza/who-influenza-recommendations/vcm-southern-hemisphere-recommendation-2023/202209_zoonotic_vaccinvirusupdate.pdf?sfvrsn=a91f123b_4 (1 January 2023, date last accessed).

[bib156] Wu NC, Wilson IA. Influenza hemagglutinin structures and antibody recognition. Cold Spring Harb Perspect Med. 2020;10:a038778. 31871236 10.1101/cshperspect.a038778 PMC7397844

[bib157] Xiong F-F, Liu X-Y, Gao F-X et al. Protective efficacy of anti-neuraminidase monoclonal antibodies against H7N9 influenza virus infection. Emerg Microbes Infect. 2020;9:78–87. 31894728 10.1080/22221751.2019.1708214 PMC6968527

[bib158] Xiong J, Zhao P, Yang P et al. Evolutionary dynamics of the H7N9 avian influenza virus based on large-scale sequence analysis. PLoS One. 2019;14:e0220249. 31404069 10.1371/journal.pone.0220249 PMC6690514

[bib159] Xiong X, Corti D, Liu J et al. Structures of complexes formed by H5 influenza hemagglutinin with a potent broadly neutralizing human monoclonal antibody. Proc Natl Acad Sci USA. 2015;112:9430–5. 26170284 10.1073/pnas.1510816112 PMC4522749

[bib160] Xiong X, Tuzikov A, Coombs PJ et al. Recognition of sulphated and fucosylated receptor sialosides by A/Vietnam/1194/2004 (H5N1) influenza virus. Virus Res. 2013;178:12–4. 24036174 10.1016/j.virusres.2013.08.007

[bib161] Yang G, Li S, Blackmon S et al. Mutation tryptophan to leucine at position 222 of haemagglutinin could facilitate H3N2 influenza A virus infection in dogs. J Gen Virol. 2013;94:2599–608. 23994833 10.1099/vir.0.054692-0 PMC4093785

[bib162] Yang H, Carney PJ, Mishin VP et al. Molecular characterizations of surface proteins hemagglutinin and neuraminidase from recent H5Nx avian influenza viruses. J Virol. 2016;90:5770–84. 27053557 10.1128/JVI.00180-16 PMC4886766

[bib163] Yao L, Chen Y, Wang X et al. Identification of antigenic epitopes in the haemagglutinin protein of H7 avian influenza virus. Avian Pathol. 2020;49:62–73. 31508993 10.1080/03079457.2019.1666971

[bib164] Yasuhara A, Yamayoshi S, Ito M et al. Isolation and characterization of human Monoclonal antibodies that recognize the influenza A(H1N1)pdm09 virus hemagglutinin receptor-binding site and rarely yield escape mutant viruses. Front Microbiol. 2018;9:2660. 30443246 10.3389/fmicb.2018.02660 PMC6222141

[bib165] Yin X, Deng G, Zeng X et al. Genetic and biological properties of H7N9 avian influenza viruses detected after application of the H7N9 poultry vaccine in China. PLOS Path. 2021;17:e1009561. 10.1371/journal.ppat.1009561 PMC8104392 33905456

[bib166] Yu F, Song H, Wu Y et al. A potent germline-like human monoclonal antibody targets a pH-sensitive epitope on H7N9 influenza hemagglutinin. Cell Host Microbe. 2017;22:471–83. 28966056 10.1016/j.chom.2017.08.011 PMC6290738

[bib167] Zhang W, Huang Q, Lu M et al. Exploration of the BF2*15 major histocompatibility complex class I binding motif and identification of cytotoxic T lymphocyte epitopes from the H5N1 influenza virus nucleoprotein in chickens. Arch Virol. 2016;161:3081–93. 27518404 10.1007/s00705-016-3013-6

[bib168] Zhang X, Chen S, Jiang Y et al. Hemagglutinin glycosylation modulates the pathogenicity and antigenicity of the H5N1 avian influenza virus. Vet Microbiol. 2015;175:244–56. 25544041 10.1016/j.vetmic.2014.12.011

[bib169] Zhang Y, Aevermann BD, Anderson TK et al. Influenza research database: an integrated bioinformatics resource for influenza virus research. Nucleic Acids Res. 2017;45:D466–74. 27679478 10.1093/nar/gkw857 PMC5210613

[bib170] Zhu X, Guo Y-H, Jiang T et al. A unique and conserved neutralization epitope in H5N1 influenza viruses identified by an antibody against the A/goose/Guangdong/1/96 hemagglutinin. J Virol. 2013;87:12619–35. 24049169 10.1128/JVI.01577-13 PMC3838140

