# Supplementary figures and images for: Epitopes in the HA and NA of H5 and H7 avian influenza viruses that are important for antigenic drift
Source: FEMS Microbiol Rev. 2024 May 11;48(3):fuae014. doi: 10.1093/femsre/fuae014 (PMC11149724; doi:10.1093/femsre/fuae014)

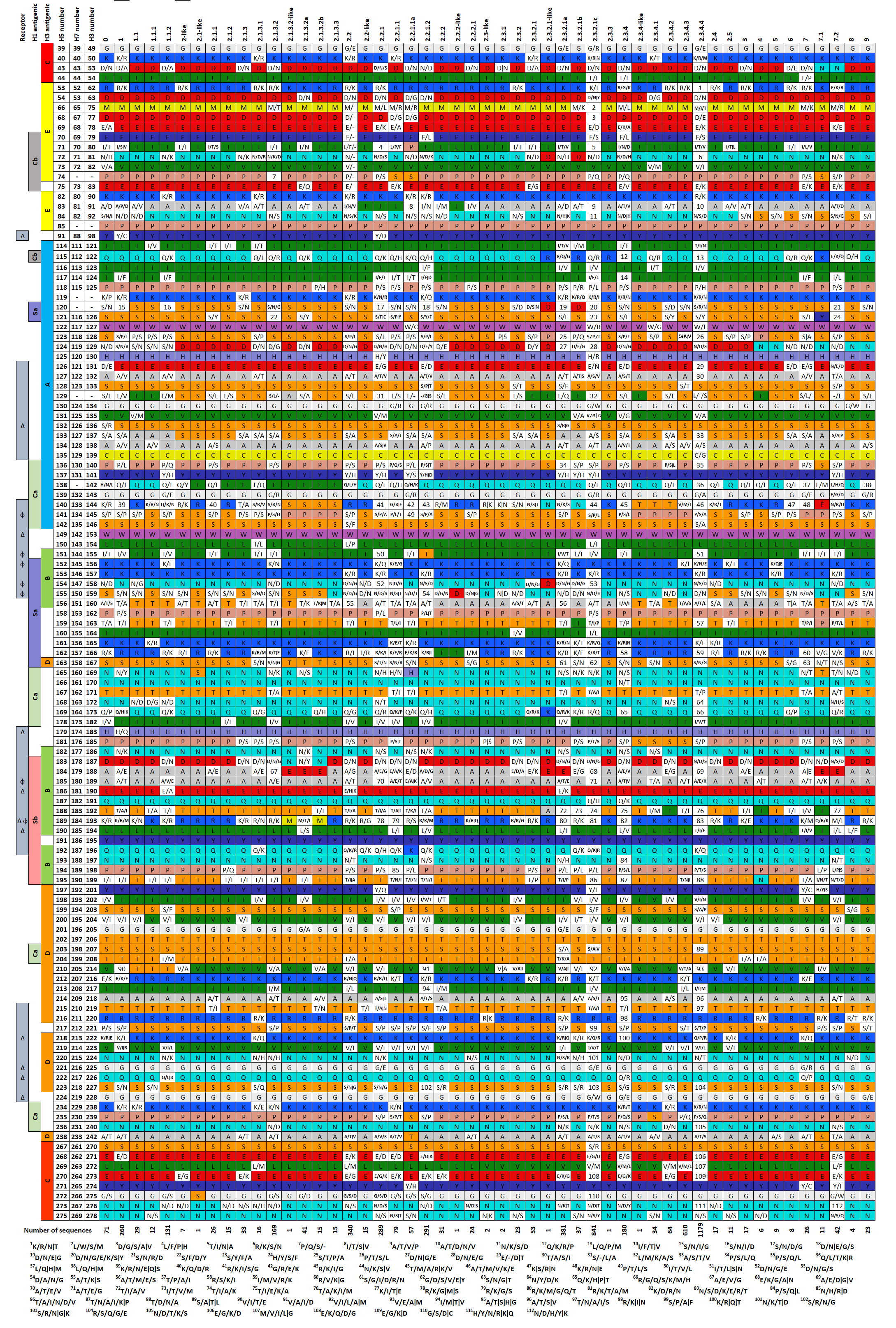

Supplement: fuae014_Supplemental_File [file fuae014_supplemental_file.zip › Figure S1.tif]
